# Rationalizing mAb Candidate Screening Using a Single Holistic Developability Parameter

**DOI:** 10.1021/acs.molpharmaceut.4c00829

**Published:** 2024-12-16

**Authors:** Leon F. Willis, Isabelle Trayton, Janet C. Saunders, Maria G. Brùque, William Davis Birch, David R. Westhead, Katie Day, Nicholas J. Bond, Paul W. A. Devine, Christopher Lloyd, Nikil Kapur, Sheena E. Radford, Nicholas J. Darton, David J. Brockwell

**Affiliations:** † School of Molecular and Cellular Biology, Faculty of Biological Sciences, University of Leeds, Leeds LS2 9JT, U.K.; ‡ Astbury Centre for Structural Molecular Biology, University of Leeds, Leeds LS2 9JT, U.K.; § The Discovery Centre—AstraZeneca PLC, Cambridge CB2 0AA, U.K.; ∥ School of Mechanical Engineering, Faculty of Engineering and Physical Sciences, University of Leeds, Leeds LS2 9JT, U.K.

**Keywords:** antibody, developability assessment, formulation, protein aggregation, kinetic stability

## Abstract

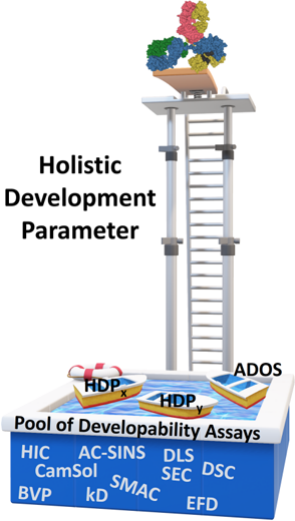

A framework for the rational selection of a minimal suite of nondegenerate developability assays (DAs) that maximize insight into candidate developability or storage stability is lacking. To address this, we subjected nine formulation:mAbs to 12 mechanistically distinct DAs together with measurement of their accelerated and long-term storage stability. We show that it is possible to identify a reduced set of key variables from this suite of DAs by using orthogonal statistical methods. We exemplify our approach by predicting the rank formulation:mAb degradation rate at 25 °C (determined over 6 months) using just five DAs that can be measured in less than 1 day, spanning a range of physicochemical features. Implementing such approaches focuses on resources, thus increasing sustainability and decreasing development costs.

## 1 Introduction

The adoption of the Quality by Design paradigm by the biopharmaceutical industry over the past two decades^1^ has led to the emergence of the concept of “developability”. This can be broadly defined as the selection of molecules with desirable biochemical and biophysical attributes, which increase the chances of translation to a commercial therapeutic manufactured at a large scale.^2−4^ Focusing on monoclonal antibodies (mAbs), many biophysical assays have been employed to probe different physicochemical characteristics of these proteins, including solubility,^5−7^ liabilities in the complementarity determining regions (CDRs),^8^ susceptibility to thermal stress,^9,10^ undesired interfacial adsorption,^11−13^ and aggregation propensity.^14−18^ Since the seminal work of Jain et al.,^4^ many groups have used Pearson^7^ or Spearman’s rank correlation^12,19^ to relate the behavior of molecules in different assays and examine the relationships between different in vitro and in silico methods.^20−23^ Nevertheless, a framework to link the outputs of these developability assessments to a chosen measurable attribute of manufacturability is lacking. The ability to do this would decrease the time for development, derisk candidate selection and scale-up, and increase sustainability, bringing enhanced provision of medicines to patients.

**Figure 1:**
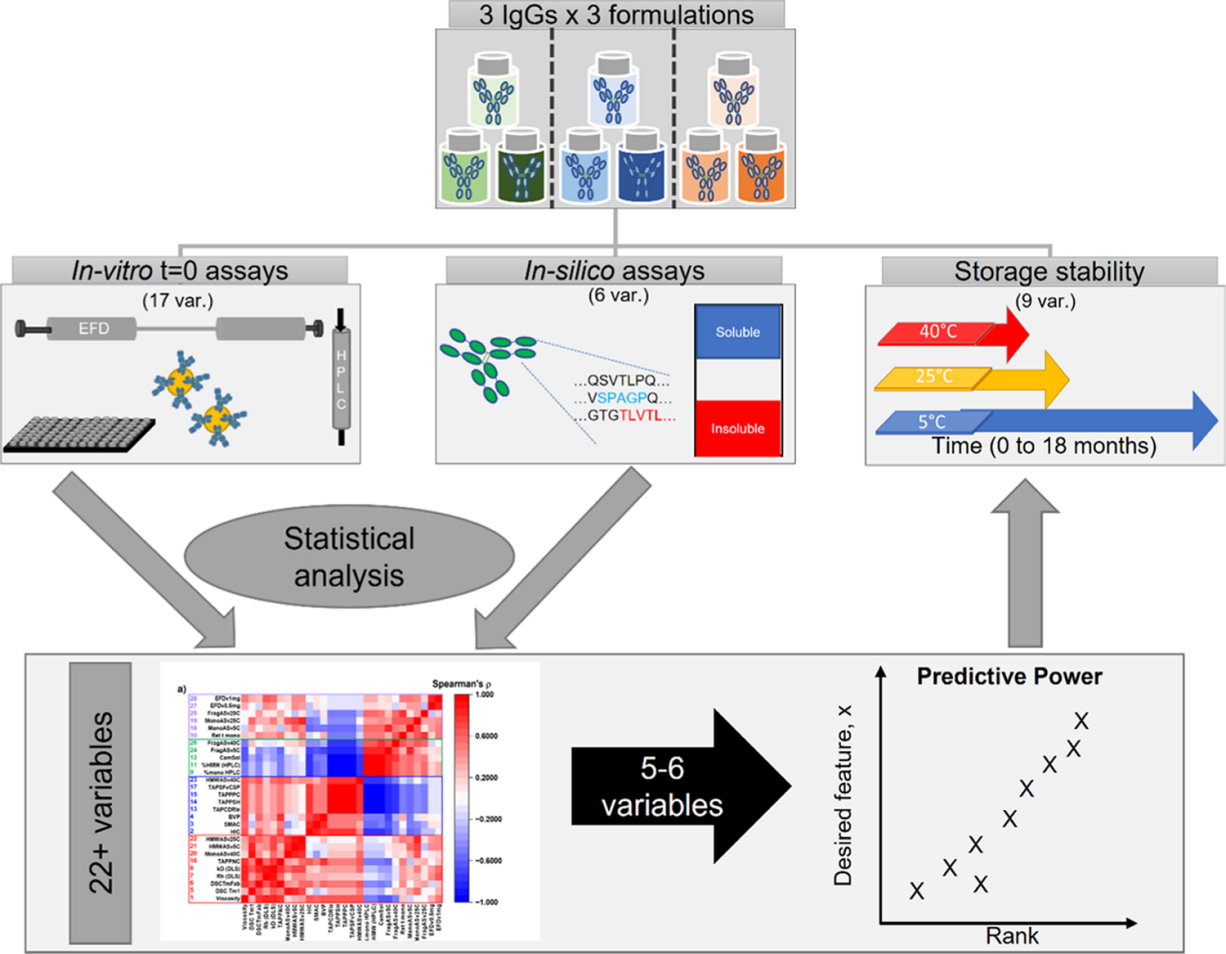
Overview of the study. Three IgG1s were placed in three different buffer conditions (histidine-arginine (green, buffer A), histidine-sucrose (blue, buffer B), and sodium citrate (brown, buffer C)), to yield nine formulation:mAb pairs. Each sample was analyzed with 10 in vitro developability assays (including the extensional flow device (EFD)) and two in silico analyses, followed by accelerated and long-term storage stability over 3 to 18 months. The completed data set (comprising at least 600 measurements) was analyzed and condensed to 32 reported assay variables, per sample. The data set was then scrutinized with an array of statistical tools. As a proof of concept, we examine whether our framework can predict the rank order of the kinetic stability of our samples at 25 °C, which is resource-intensive in terms of both time and material, using less resource-intensive “time equal zero” assays.

To address this issue, here we describe a logical framework to condense the outputs of a focused set of developability assays (DAs) to a single parameter. This parameter, derived from assays employed early in development, has the predictive power of a user-defined measurable attribute of manufacturability (Figure 1). To do this, we obtain a data set derived from 12 mechanistically distinct DAs (with the outputs captured by 23 variables) including in silico analyses on three IgG1s in three formulations and complement these with long-term and accelerated stability data obtained in the same buffers (captured by nine variables). As accelerated and long-term (i.e., real-time) degradation rates are universal, yet expensive-to-determine quality attributes essential within the regulatory framework (for a typical mAb, this takes over two years and consumes grams of material),^24^ we chose the kinetic stability of the samples at 25 °C as our measurable attribute of manufacturability.

Statistical analysis of the data set shows that the DAs are grouped into four families that probe distinct biophysical features which can be used to rank formulation:mAbs holistically. We then show that a combination of suitably scaled outputs from a focused, nondegenerate set of DAs that probe multiple biophysical attributes can be used as an indicator of kinetic stability early in the development pipeline by predicting relative storage stability at 25 °C. The general methodology (which could be applied to other manufacturing attributes of the user’s choosing) is rapid and resource-efficient, and its ability to capture storage stability derisks and increases the sustainability of early-stage candidate selection.

## 2 Experimental Section

### 2.1 Antibodies and Formulations

All antibodies were expressed in an IgG1 format in CHO cells and purified from the culture medium using Protein A chromatography.^25^ Each IgG1 (mAb1, mAb2, and mAb3) was then dialyzed into the following formulations: 20 mM l-His, 190 mM l-arginine, pH 6 (formulation A); 20 mM l-His, 220 mM (7.5% w/v) sucrose, pH 6 (formulation B); and 25 mM sodium citrate, pH 5.0 (formulation C) by repetitive buffer exchanges using Millipore Centricon 30 000 MWCO filters, according to the manufacturer’s protocol. Briefly, the tubes were primed with 15 mL of the new formulation buffer and centrifuged at 3500*g* for 10 min. The sample was loaded into the tubes and centrifuged as before for 30 min. The filtrate was discarded, and the retentate was diluted to 15 mL with formulation buffer. This process was repeated at least 5 more times until the final desired concentration and volume were reached.

Protein concentration was determined at 280 nm by using a Trinean DropSense96 UV–vis spectrophotometer. Samples were diluted to a final concentration of 50 mg/mL, then syringe-filtered through a 0.22 μm filter (Millipore) in a laminar flow hood. 10% (w/v) PS80 was added to each mAb/formulation to a final concentration of 0.02% (w/v) and then refiltered under sterile conditions (0.22 μm), then vialed in 1.1 mL aliquots using 2R glass vials, rubber stoppers, and crimp sealed. One set of vials was frozen at −80 °C, for use later in HIC, SMAC, and EFD assays. The osmolality and pH of the samples were measured using an OsmoPro and Mettler Toledo pH meter, respectively, to confirm the formulations were within specification (see the Supporting Information).

For reference, this yields three IgGs in three formulations (nine samples in total), with the code names displayed in the unshaded boxes in Table 1.

**Table 1:** Nomenclature for IgGs and Buffer Conditions Used

formulation:mAb	mAb1	mAb2	mAb3
formulation A (His-Arg)	A1	A2	A3
formulation B (His-sucrose)	B1	B2	B3
formulation C (Na citrate)	C1	C2	C3

### 2.2 Developability Assays

Methods for the rheology of (surfactant-free) formulations, HIC, SMAC, BVP-ELISA, AC-SINS, DSC, DLS, BMI, CamSol, TAP, and soluble protein concentration measurements, are provided in the Supporting Methods.

### 2.3 Extensional Flow Device and HPLC Assay

Design and operation details of the extensional flow device (EFD) can be found elsewhere.^16,26−28^ The current work used a modified version of the original device, with a 3D-printed insert allowing three pairs of 1 mL Gastight Hamilton syringes to be mounted and driven simultaneously. Each EFD experiment initially begins with 3× buffer-rinsed syringes fitted with fresh 75 mm long, 0.3 mm i.d. borosilicate glass capillaries (Sutter Instruments) via ferrule compression fittings (Hamilton) and Gilson P10 O-rings. The mAb solutions were prepared from thawed vials of each respective formulation. The aliquot was diluted ∼10-fold in its respective formulation buffer, syringe-filtered (0.22 μm, Millipore), and the concentration was determined (after a further 20-fold dilution) by UV–vis spectroscopy (Shimadzu UV-1800). 0.5 mL portion of protein solution (0.25-, 0.5-, and 1 mg/mL) was drawn into each respective sample syringe, removing visible air bubbles prior to connection to their buffer-rinsed “receiver” syringe. The syringe pairs were fixed with top-mounted 3D-printed clamps, before being driven by a linear stage using a stepper motor at a velocity of 8 mm/s for a defined number of passes (10–500). The pass conditions were controlled by a microprocessor and a visual display. Once finished, syringes were disassembled and the solutions were slowly placed into fresh Eppendorf tubes and kept on ice. Control samples were incubated ambiently alongside the 500 passes samples (which takes ∼50 min to complete) at each concentration. The syringes were washed with 2% (v/v) Hellmanex-III (aq), Milli-Q water, and formulation buffer prior to each new experiment.

To quantify EFD-induced aggregation, samples were clarified by ultracentrifugation, spinning 2 × 150 μL of each sample for 30 min at 30 000 rpm (TLA100 rotor, Beckmann Coulter). 2 × 100 μL of supernatant was removed from each respective sample tube, with the supernatants then combined and loaded in a 300 μL conical insert polypropylene vial (VWR), before crimp-sealing with PTFE/Aluminum lids (Thermo Fisher). Samples were analyzed by HP-SEC on a Shimadzu Nexera LC-40 system. 20 μL of sample was injected onto a TOSOH G3000swxl column, eluting isocratically with HP-SEC mobile phase (0.1 M sodium phosphate dibasic, 0.1 M sodium sulfate pH 6.8), at a flow rate of 0.5 mL/min. Following detection at 280 nm with a PDA detector, the chromatograms were integrated in LabSolutions software, and % monomer remaining was calculated by normalizing the peak areas to those of the respective, quiescent control samples. The observed rate of monomer loss was computed by using the SLOPE function in Microsoft Excel.

### 2.4 Accelerated (AS) and Long-Term Storage Stability Study

This study commenced in January 2020. Boxes containing vials of A1–C3 were placed in incubators at the temperatures and for the durations stated in Table 2.

**Table 2:**
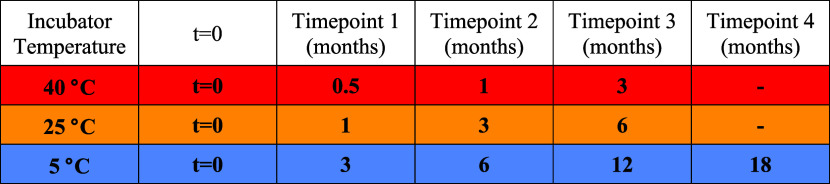
List of Samples Taken for Analysis from the Stability Study

Due to the COVID-19 pandemic, all time point samples (apart from 0-, 0.5-, and 1-month samples) were pooled and stored at −80 °C prior to their quantification in April 2022. To quantify the species remaining in solution at each time point, samples were diluted 1:4 in PBS (Sigma) into a 0.45 μm centrifugal filter unit (Millipore) and spun at 16 700*g* for 1 min. Alongside the formulated samples, a series of standards (PBS, HP-SEC mobile phase (0.1 M sodium phosphate dibasic, 0.1 M sodium sulfate pH 6.8), Nip228 reference standard IgG (in 20 mM l-His, 240 mM sucrose pH 6.0), and BioRad column calibrants) were clarified in the same fashion.

A 2 × 25 μL sample was injected onto a TOSOH G3000swxl column, equipped with a guard column. The samples were eluted isocratically in the HP-SEC mobile phase at 1 mL/min on an Agilent HPLC system. Peak areas were quantified by integration using ChemStation software. The monomer peak was considered as the major peak with a retention time of ∼8.3 min. Any peaks detected with a shorter retention are higher-molecular-weight species (HMW). Any peaks that elute after the monomer are considered to be fragments. The area values were input into Excel, including the standards, which passed internal validation levels. After averaging the technical replicates, the SLOPE function was used to determine the observed relative rates of % change in monomer, HMW content, and % fragment over the respective time courses above. We thus highlight that all of the observed rates pertaining to the kinetic stability study are relative observed rates, but we omit “relative” for brevity throughout the manuscript.

Finally, the error on the observed rate of change in % monomer at 25 °C test data set was calculated by including the error (s.d.) from both technical replicates for each formulation. The average coefficient of variation from the SLOPE analysis (for the entire data set = 0.036%/month) was used as a default value where samples had zero error. Instrumental error weighting was used (1/*CV*^2^) and a linear fit (*y* = *a* + *bx*) was performed in OriginPro to obtain the gradient (*b*) and standard error (from the fit) for each formulation (Figure 3). This analysis was also performed on the 5 °C (average SLOPE *CV* = 0.074%/month).

### 2.5 Statistical Analyses

Data were processed in Microsoft Excel. Correlation analysis and Hierarchical Clustering of Spearman correlation coefficients were performed in OriginPro 2023b. For the clustering, Euclidean distances and group average clusters were used to draw the dendrogram. All graphs in the manuscript were plotted in this software. Details on Multiple Linear Regression (OriginPro 2023b) are detailed in the Supporting Methods.

### 2.6 Ranking and Sensitivity Analysis

Assay variables were ranked in Microsoft Excel using the RANK.AVG function. Values were ranked from the most desirable to least desirable value, depending on the favorable direction of the assay, e.g., a high Tm,app is desirable, while a low Tm,app is undesirable.

For the sensitivity analysis, one formulation, e.g., A1, was removed from the data set, the data reranked as above, and Hierarchical Clustering was performed as stated in the main text. This process was repeated sequentially for each formulation. The least significant correlations are flagged in OriginPro, generally pertaining to branches within each assay group that have the largest distance from the baseline, e.g., variables 30 and 32 in Figure S19. To further evaluate the robustness of the assay variable groupings, the approach of Lu et al. was employed.^29^ The total number of times a variable paired with its immediate neighbor was counted in each iteration of the analysis, then divided by the total number of iterations (10 in this case). Values (termed P) close to 1 reflected the most robustly clustered variables (Figure S21).

### 2.7 Averaging of Developability Output Score (ADOS) Algorithm

The foundations for the following analysis can be found elsewhere.^4,16^ First, the scores for a formulation, *i*, in an assay variable, *j*, were scaled according to their position within the distribution of the observed data (eq 1)
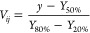
1where *V*_*ij*_ = scaled value, *y* = reported assay value, *Y*_50%_ = median, *Y*_80%_ = 80th percentile value, and *Y*_20%_ = 20th percentile value.

Next, the scaled values were normalized onto a best (0) to worst (1) scale (eq 2a)
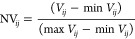
2awhere NV_*ij*_ = normalized scaled value, min* V*_*ij*_ = smallest scaled value for the assay variable, and max* V*_*ij*_ = largest scaled value for the variable.

For assay variables where the smallest number corresponds to the worst score, e.g., the formulation:mAb with the lowest Tm_app_ has the poorest thermal stability and a very negative monomer-loss slope reveals faster aggregation or degradation, the scores were adjusted with eq 2b.
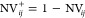
2bwhere NV_*ij*_^+^ = adjusted normalized scaled value.

Next, using the groups from Figure 4b, identified by Hierarchical Clustering, the average score for a formulation across each group was calculated (eq 3).
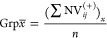
3where Grp*x̅* = averaged formulation score within assay group *x*, (∑NV_*ij*_^(+)^)_*x*_ = sum of adjusted/normalized scaled values within assay group, and *x* and *n* = number of assay variables in group *x*. For example, Group 1 (red group, Figure 4) has nine variables, thus *n* = 9 for this group.

Finally, using the approach of Jain et al.,^4^ a “distance from ideal” was calculated for each formulation (eq 4), which we term the averaged developability output score (ADOS).
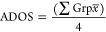
4where ADOS = distance from ideal for each formulation and 4 = number of assay groups.

By using this algorithm, and then ranking the ADOS values on a best (lowest) to worst (highest) scale, formulations that obtain low ADOS values across the assay groups are closer to “ideal” than those that obtain high values. Formulations with a high ADOS can thus be more confidently deemed suboptimal. The values obtained from eq 3 can be weighted by Multiple Linear Regression to obtain ADOS_MLR_ (see Supporting Information, including Figure S23).

### 2.8 LASSO Regression on Ranked Data

LASSO regression was initially performed to identify the minimal set of assays required to predict the observed rate of change in % monomer at 25 °C. This was performed on the ranked data using an XLSTAT 2023. This method is independent to and has a different mathematical basis to the MLR approach and is ideally suited to data sets where there are more variables than data points.^30^ The 19 ranked assay variables (shown in Tables 3 and 4) were initially correlated against the ranked rate of monomer loss at 25 °C, using cross-validation to find the regularization parameter, λ, using the default settings (5-fold, 100 λ values) (Figure S24). This analysis was subsequently repeated using the 19 variables above or all of the variables from Tables 3 and 4 (including those in bold) to generate a predictive algorithm. An inherent strength of LASSO is that it identifies only those variables that are important for the resulting model (Figure S24).

**Table 3:**
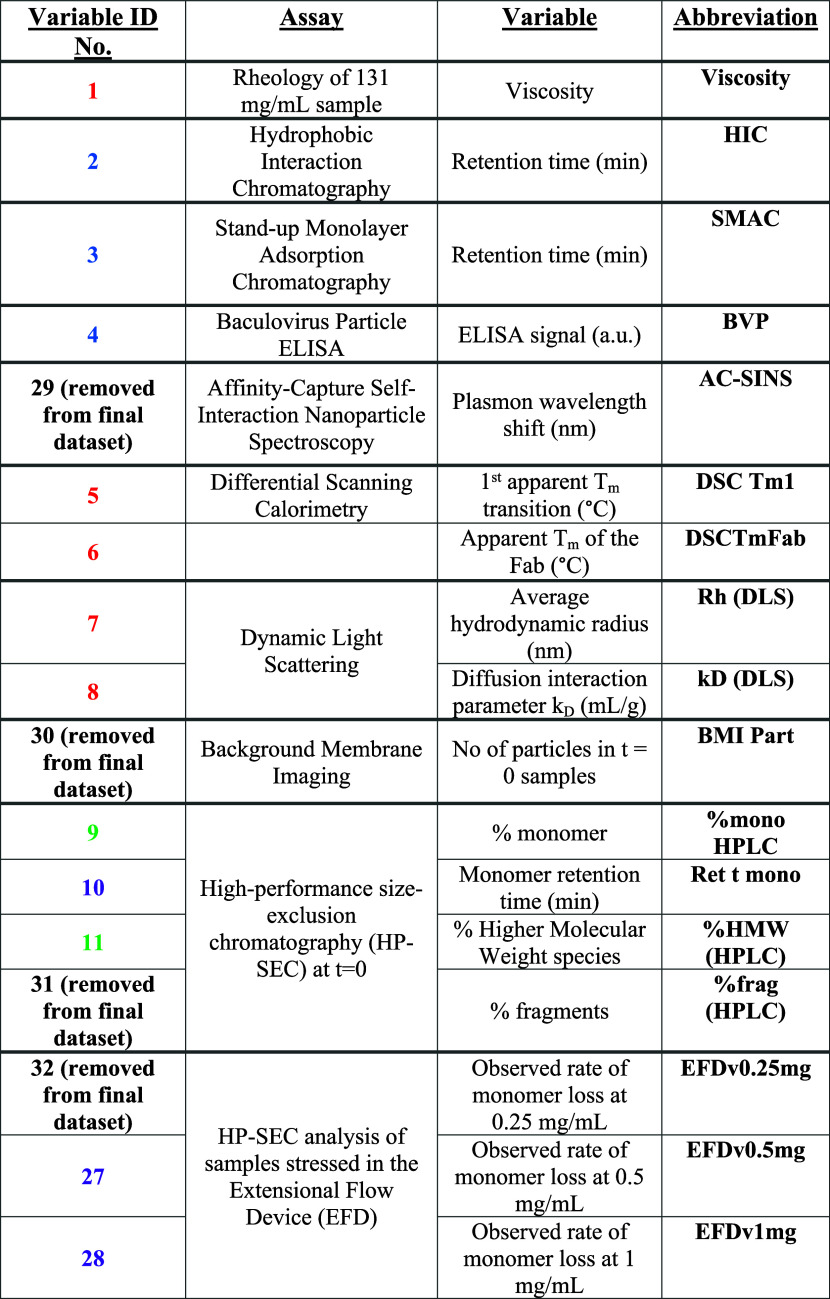
Summary of the Variables Output by the DAs (Developability Assays) Employed at *t* = 0 on the Formulation:mAb Panel^a^

a The colors of the variable ID number correspond to the family tree group color in Figure 4. Variables with ID numbers in bold were deemed difficult to cluster and were removed from the final clustering data set in Figure 4 (see Figure S19).

**Table 4:**
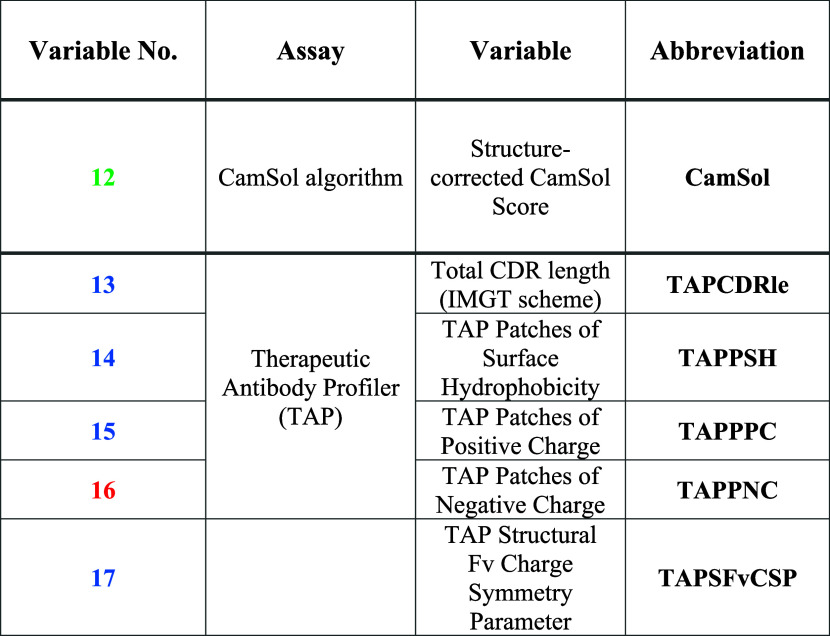
Summary of the Variables Output by the In Silico Assays Employed on the Variable Domain Sequences/Homology Models of mAb1, mAb2, and mAb3^a^

a The colors of the variable ID number correspond to the Family Tree Group color in Figure 4.

## 3 Results

### 3.1 Assessing the Developability and Kinetic Storage Stability of a Panel of Antibody Formulations

The formulation:mAb panel comprised three IgG1s: mAb1, mAb2, and mAb3 in three different buffers selected to reflect typical marketed product formulation compositions^31^ (20 mM l-His +190 mM l-Arg pH 6.0 (Buffer A), 20 mM l-His +7.5% (w/v) sucrose, pH 6.0 (Buffer B), and 25 mM sodium citrate pH 5.0 (Buffer C)). The mAbs were dialyzed into these buffers, diluted to a final concentration of 50 mg/mL, spiked with 0.02% (w/v) polysorbate 80 (PS80), and vialled (Methods section), yielding nine formulation:mAb samples, A1–C3 (with the letter identifying the buffer and the number the mAb identity, e.g., B2 is mAb2 in Buffer B (His-sucrose), Table 1, Methods section).

Each of the nine formulation:mAbs were initially characterized using 10 different DAs (Figure 1, Table 3, Methods section, and Supporting Information). These were selected to characterize a broad array of different biophysical features as evidenced by their inclusion in different branches of hierarchical clusters of DAs reported by Jain et al.^4^ or, for assays not included in the Jain study, their published ability to provide additional insight or prediction of mAb developability (e.g., Diffusion interaction parameter (*kD*) and the EFD, see below). The assays, grouped by the biophysical property being probed and the number of output variables measured by each technique, are briefly described below and more fully (together with an identification number used herein) in Table 3. Group (I) probes Colloidal stability: viscosity of the concentrated, surfactant-free formulations (yielding 1 variable (var.) output), retention times in size exclusion (SEC), hydrophobic interaction (HIC), and stand-up monolayer adsorption chromatography (SMAC) (each yielding 1 var.), affinity capture, self-interaction nanoparticle spectroscopy plasmon wavelength shift (AC-SINS) (1 var.), and dynamic light scattering (yielding 2 var., the hydrodynamic radius and the *kD*). Group (II) probes thermal stability by differential scanning calorimetry (DSC) (2 var. the first and apparent Fab melting temperature) while Group (III) probes miscellaneous features of the molecules: Baculovirus particle adsorption, linked to rapid in vivo clearance^32^ (BVP) (1 var.), the number of subvisible particles present by background membrane imaging (BMI) (1 var.) and, finally, the rates of monomer loss induced by the EFD at 0.25-, 0.5-, and 1 mg/mL (3 var.). This device, developed at Leeds,^16,26−28^ subjects proteins to the potentially synergistic stresses of hydrodynamic flow fields and interfaces that are experienced by proteins throughout their manufacture, including depth filtration and fill-finish steps.^33^ The EFD provides unique insight relative to other assays,^16,26,27^ suggesting its utility as a complementary DA to those commonly employed by the biopharmaceutical industry.^16^ The use of this assay is explained in detail in the Methods section. These experimentally derived variables were augmented with further variables (Group (IV)), derived from in silico methods (Table 4): prediction of CDR and F_V_ liabilities using Therapeutic Antibody Profiler^8^ (5 var.) and the structure-corrected solubility of the variable domains using CamSol^5^ (1 var.).

**Figure 2:**
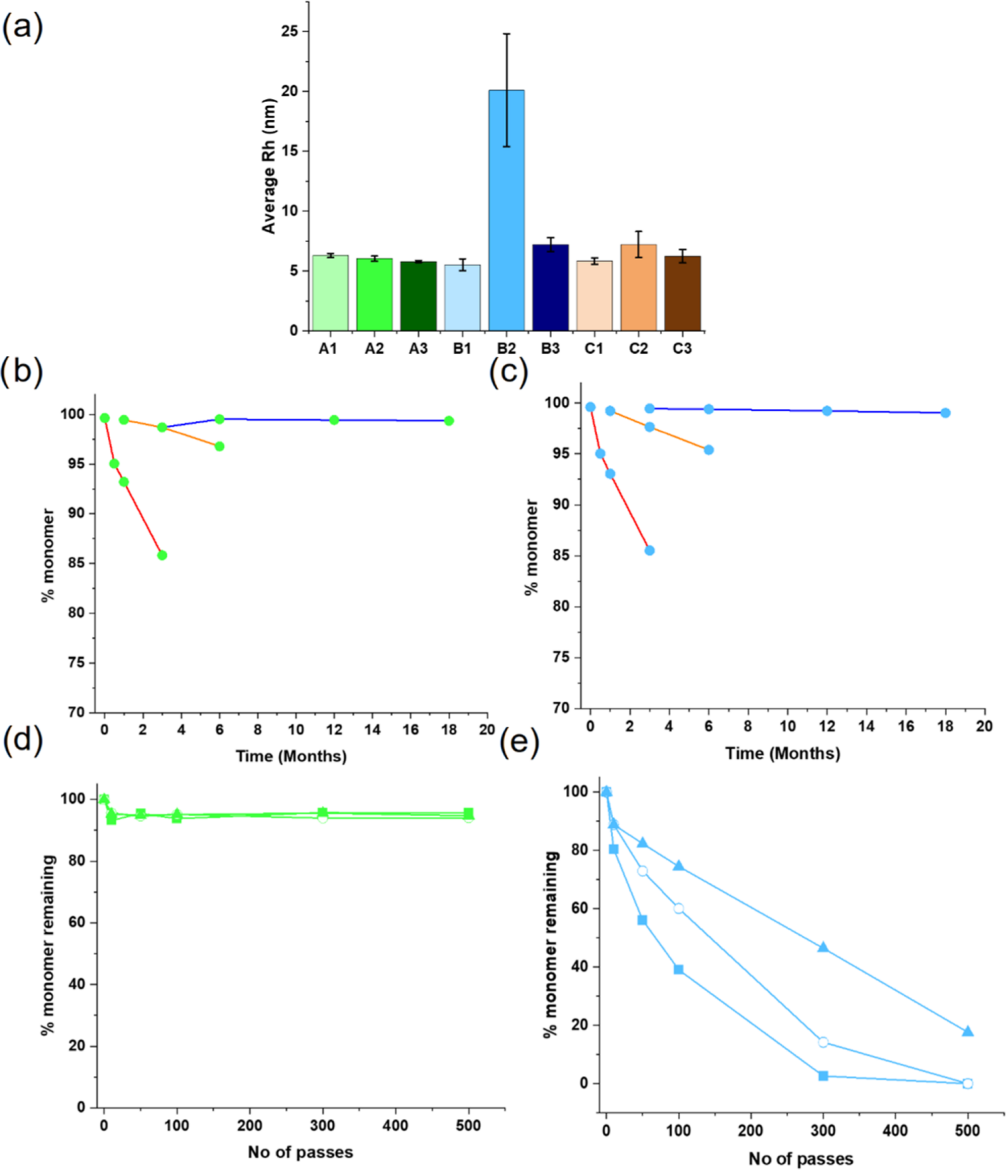
Using the “developability toolkit” to screen antibody formulations. (a) Average hydrodynamic radius (*R*_h_) of the nine samples in the study, obtained using dynamic light scattering, measured at concentrations between 2 and 20 mg/mL (Supporting Methods and Figure S5). Bars are colored according to the formulation:mAb, error bars = s.d. (b, c) HP-SEC analysis of formulation:mAbs A2 (mAb2 in histidine-arginine) (b) and B2 (mAb2 histidine-sucrose) (c) following accelerated and long-term storage stability (Methods section). Samples were incubated at 50 mg/mL for the times and temperatures indicated, with the relative % monomer in the HP-SEC trace quantified. Lines through the points at 5 °C (blue), 25 °C (orange), and 40 °C (red) are a guide to the eye, not fit to the data. (d, e) HP-SEC analysis for formulation:mAbs A2 (d) and B2 (e) following stress in the EFD (Methods section). Initial [protein] in EFD experiments = 0.25 mg/mL (squares), 0.5 mg/mL (open circles), and 1 mg/mL (triangles), with % monomer remaining quantified by HP-SEC (Methods section).

Exemplar data, together with violin and box plots for all nine formulation:mAbs, are shown for each DA in Figures S1–S12 together with a description of each assay (Supporting Methods). Generally, most DAs produced non-normally distributed populations with long tails, as observed previously^4,16^ and the relationship between DA outputs is often difficult to rationalize. For example, the viscosity of formulation:mAb B2 was four times above the upper limit typically acceptable for prefilled syringe administration^34^ (Figure S1) and showed evidence of aggregation by DLS (Figure 2a). Despite this, in silico analyses failed to flag liabilities in the variable domains of this and the other mAbs which could lead to colloidal instability (Figures S7 and S8).

To obtain kinetic stability data for each of the characterized formulation:mAbs, we incubated the vials under accelerated stability (AS) conditions at 40 °C, as well as long-term storage (LTS) conditions at 25 and 5 °C. Vials were removed from the incubators after: 2 weeks (0.5 months), 1 month, and 3 months at 40 °C; 1, 3, and 6 months at 25 °C; and 3, 6, 12, and 18 months at 5 °C (Methods section). While HP-SEC was used to quantify the relative amount of monomeric, higher-order (high molecular weight, HMW), and fragmented mAbs injected onto the column, the total soluble protein concentration was additionally quantified using UV–visible spectroscopy with 350 nm correction to remove scattering artifacts (Supporting Methods, Figure S13). Together, these analyses showed that the majority of samples formed soluble HMW species and fragments over the course of the AS and LTS studies but formulation:mAbs C2 and C3 formed insoluble aggregates after incubation for 3 months at 40 °C (Figure S13(biii,ciii), respectively), resulting in the removal of these points from the observed rate of monomer-loss analysis.

**Figure 3:**
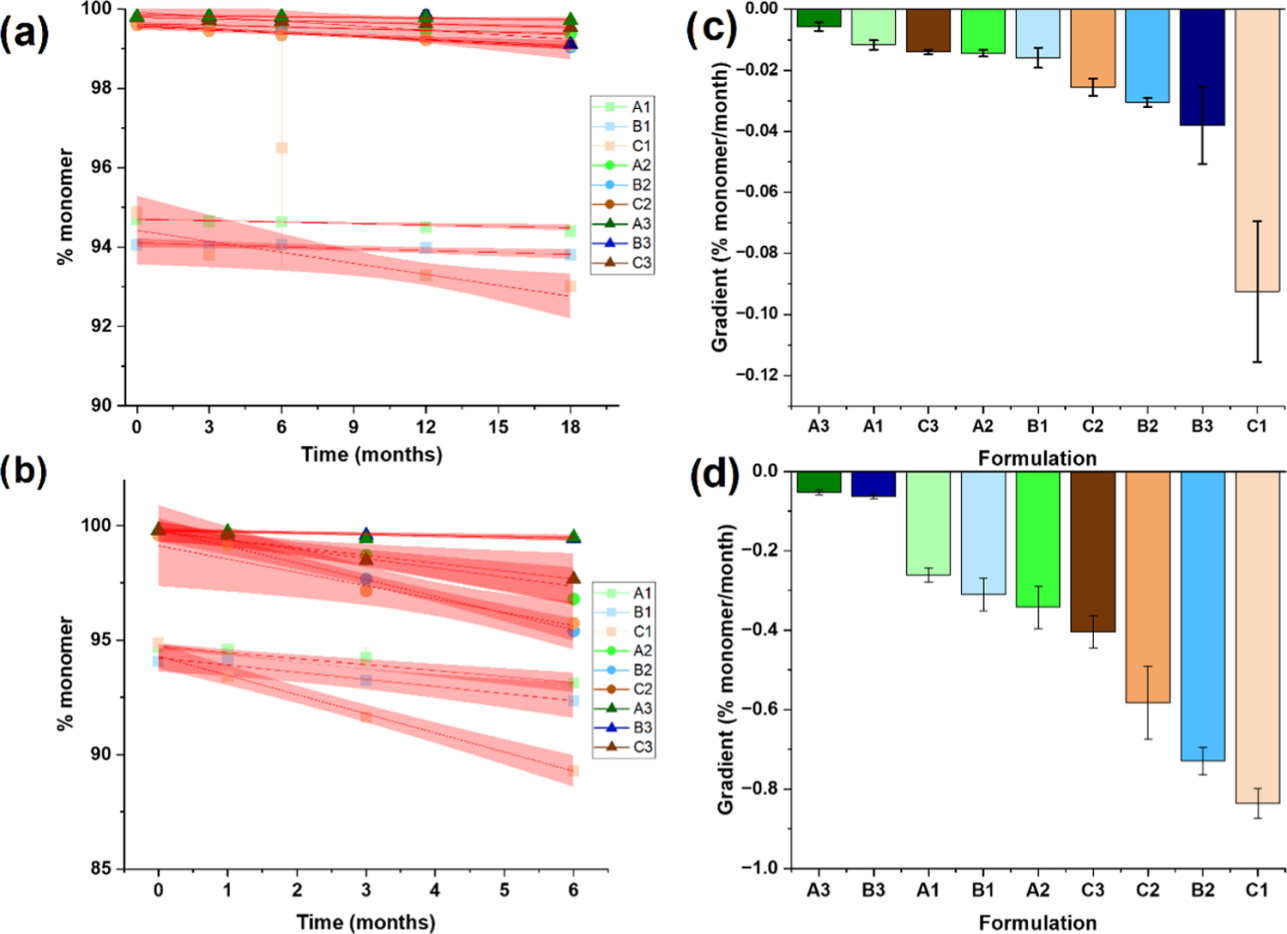
Change in % monomer over 6 to 18 months at 25 and 5 °C, respectively. % monomer calculated for formulation:mAbs A1–C3, derived from technical repeats at 5 °C (a) and 25 °C (b). Fitting a straight line to the data yields the observed rate (gradient) alongside a standard error. Red region = 95% confidence interval. Observed rates for the nine formulation:mAbs at 5 °C (c) and 25 °C (d); error bars = standard error.

The observed rates of change in % monomer, HMW species formation, and fragmentation (quantified by HP-SEC,^35^Methods section), for each formulation at each temperature, were calculated using linear regression (Methods section). These data are shown in the Supporting Information (Figures S14–S17) comprising Group (V) in our suite of DAs (Table 5). A decrease in the amount of monomer was accompanied by a concomitant increase in the HMW species and fragments detected within each sample (Figures S14–S17). Generally, incubation at higher temperatures accelerated monomer loss for all of the mAb samples (Figure 2b,c, for example), with these rates becoming 30 and 200 times slower at 25 and 5 °C, respectively (based on the median rate for all nine formulation:mAbs, Figure S18). In accordance with other studies,^36−38^ this process cannot be described by simple Arrhenius kinetics,^36,37^ obviating the use of recently developed kinetic models^39−41^ to predict the LTS/shelf life for these formulation:mAbs. Under the conditions and buffers used here, all formulation:mAbs showed minimal degradation at 5 °C (ca. 0.01–0.09% monomer/month, Figure 3) precluding the use of these data as our metric of manufacturability, given the relative size of the experimental and fitting error compared to the data points (average coefficient of variation = 0.074%/month, median rate of loss = 0.015%/month, Section 2.4). By contrast, degradation rates were approximately 10 times faster at 25 °C (Figure 3), and consequently, these data were used to rank formulations as the error (0.036%/month) was far smaller than the measured rate of loss (median rate of loss = 0.34%/month). We note here that at 25 °C formulation:mAbs C2, B2, and C1 exhibit statistically significantly different rates to each other and also to A3 and B3 (which exhibit indistinguishable rates) and A1, B1, A2, and C3 (which display varying difference in significance to each other but are distinct to A3 and B3 and C2, B2 and C1). For simplicity, we first describe our analyses using a ranking based on the observed rate values (i.e., left to right in Figure 3d ranks formulation:mAbs from best to worst). We then show how changing the ranks for A1, B1, A2, and C3 has minimal effect on the resulting outputs, validating the use of this data set as our test example for the statistical workflow presented herein.

**Table 5:**
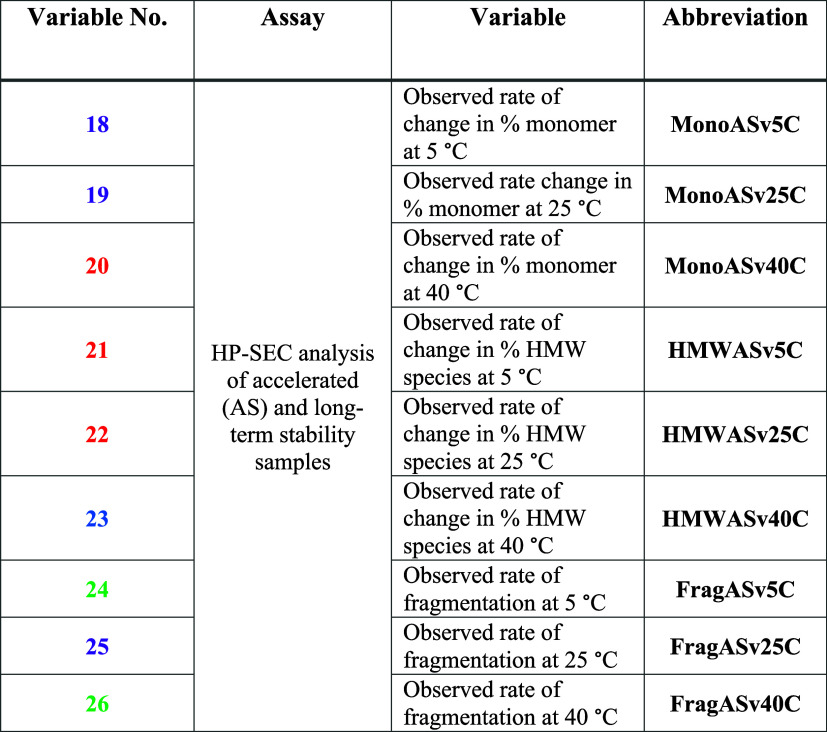
Summary of the Variables Output from the Accelerated and Long-Term Stability Study on the Formulation:mAb Panel^a^

a The colors of the variable ID number correspond to Family Tree Group color in Figure 4.

### 3.2 Statistical Analysis Reveals the Relationships between the Developability Assay Variables

The first aim of this study was to determine the relationship between the outputs of each variable, obtained from the suite of DAs used, to allow the selection of a reduced set of complementary, nondegenerate DAs. To do this, we performed Spearman’s rank analysis of the variables, followed by Hierarchical Clustering of the resulting correlation coefficients, as described in previous studies.^4,12,16,19^ As more than one variable can be obtained from some of DAs used here (e.g., the hydrodynamic radius (*R*_h_) and diffusion interaction parameter (*kD*) are both obtained from DLS), a total of 32 assay variables for each of the nine formulation:mAbs (referred to as samples herein) were analyzed (see Tables 3–5, Figure 1), generating a Spearman’s rank correlation coefficient for each pairwise variable combination. These values were then subjected to Hierarchical Clustering analysis, as described previously^4,16^ (Figure S19a), yielding six branches each containing variables that are related by the information they provide (Figure S19b). To better understand the strength of the clustering, the least significant (longest distance from baseline) assay variable in each branch was noted (Supporting Methods). Following this, the data obtained for each formulation:mAb (A1–C3) was iteratively removed from the panel and the analyses described above were repeated. Repeating this process for the remaining nine combinations of samples (i.e., the data set comprising all formulations plus nine data sets with one formulation:mAb removed from each) allowed the identification of variables that clustered poorly with other assays. Using this approach, four variables, approximately equivalent to removing one formulation:mAb, were found to be the least significant branch assay in at least six dendrograms in the analysis, suggesting that these variables were distinct in the information they provided. As the first aim of this study was to understand degeneracy within DAs, these variables (AC-SINS (var. 29), BMI (var. 30), initial levels of fragmentation by HP-SEC (var. 31) and the observed rate of EFD-induced monomer loss at 0.25 mg/mL (var. 32)) were removed from the analysis.

**Figure 4:**
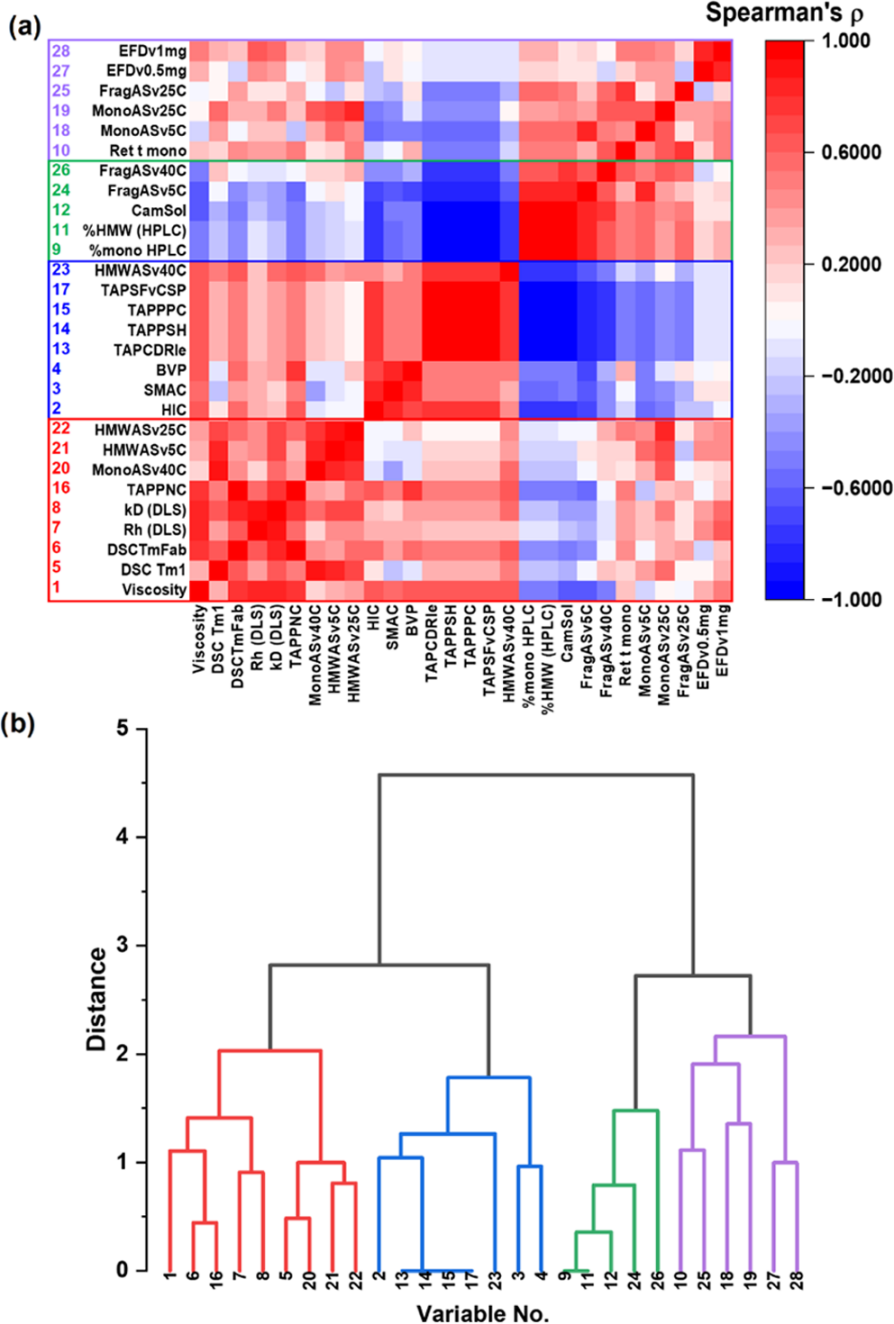
Statistical analysis clusters assay variables in the developability “assay pool”. (a) Heatmap of Spearman’s rank correlation coefficients (ρ) for the pairwise interactions between the 28 best-clustered assay variables in the data set. (b) Hierarchical clustering analysis of these variables generates a “Family Tree” comprising four branches of related assays. The observed rates of monomer loss after stress in the EFD at 0.5 and 1 mg/mL (variables 27 and 28, respectively) are in the same branch (purple) as the equivalent rates following storage stability at 5 and 25 °C (observables 18 and 19, respectively). The assays from which the variables are derived and their abbreviations are listed in Tables 3–5.

Hierarchical Clustering of the pairwise Spearman’s correlation coefficients was repeated on the remaining 28 variables obtained from 10 DAs (Tables 1–3) for the nine formulation:mAbs and identified four branches of related assay variables (Figure 4a,b). The red cluster is the largest, comprising nine variables (variables 1, 5–8, 16, and 20–22), probing several molecular features including the viscosity (variable 1), thermal stability (variables 5 and 6), and observed rate of monomer loss at 40 °C (variable 20). The relatedness of these latter two assays makes mechanistic sense: poor thermal stability may result in the promotion of unfolding and aggregation via the unfolded state at elevated temperatures.^42,43^ The blue cluster (8 variables) comprises many of the TAP metrics (variables 13–15 and 17), as well as measures of molecular “stickiness”^44^ (HIC, SMAC, and BVP, variables 2–4). The smallest green cluster of five variables (9, 11, 12, 24, and 26) probes miscellaneous features, including the observed rate of fragmentation at 40 °C (variable 26). The final purple cluster contains six variables (variables 10, 18, 19, 25, 27, and 28). Notably, this includes the observed rates of monomer loss at 5 and 25 °C (variables 18 and 19, respectively) which stem from the same branch, as do the observed rates of monomer loss in the EFD at 0.5- and 1 mg/mL (variables 27 and 28, respectively). The robustness of these relationships was further assessed by sequentially removing the data obtained from each formulation:mAb from the data set, which was then reranked and reanalyzed (example dendrograms in Figure S20).

To quantify the differences in the dendrograms, we used the approach of Lu et al.^29^ and calculated the frequency with which an assay variable paired with its immediate neighbors over all iterations, with a median “*P*-value” of 0.9 (*P*-values range from 1 (no change in pairing) to 0 (all pairings changed), Figure S21). For reference, 10 of 28 variables did not change pairing at all, with 11 of 28 changing 1 or 2 times (Supporting Methods and Figure S21). At a coarser level, an assay was found to be assigned to a different group (branch) only 5 of 28 times (median *P* = 1). Small changes in assay groupings for subsets of antibody samples have been observed previously.^4,16^

### 3.3 Developability Assay Outputs Can Be Condensed into a Single Metric

**Figure 5:**
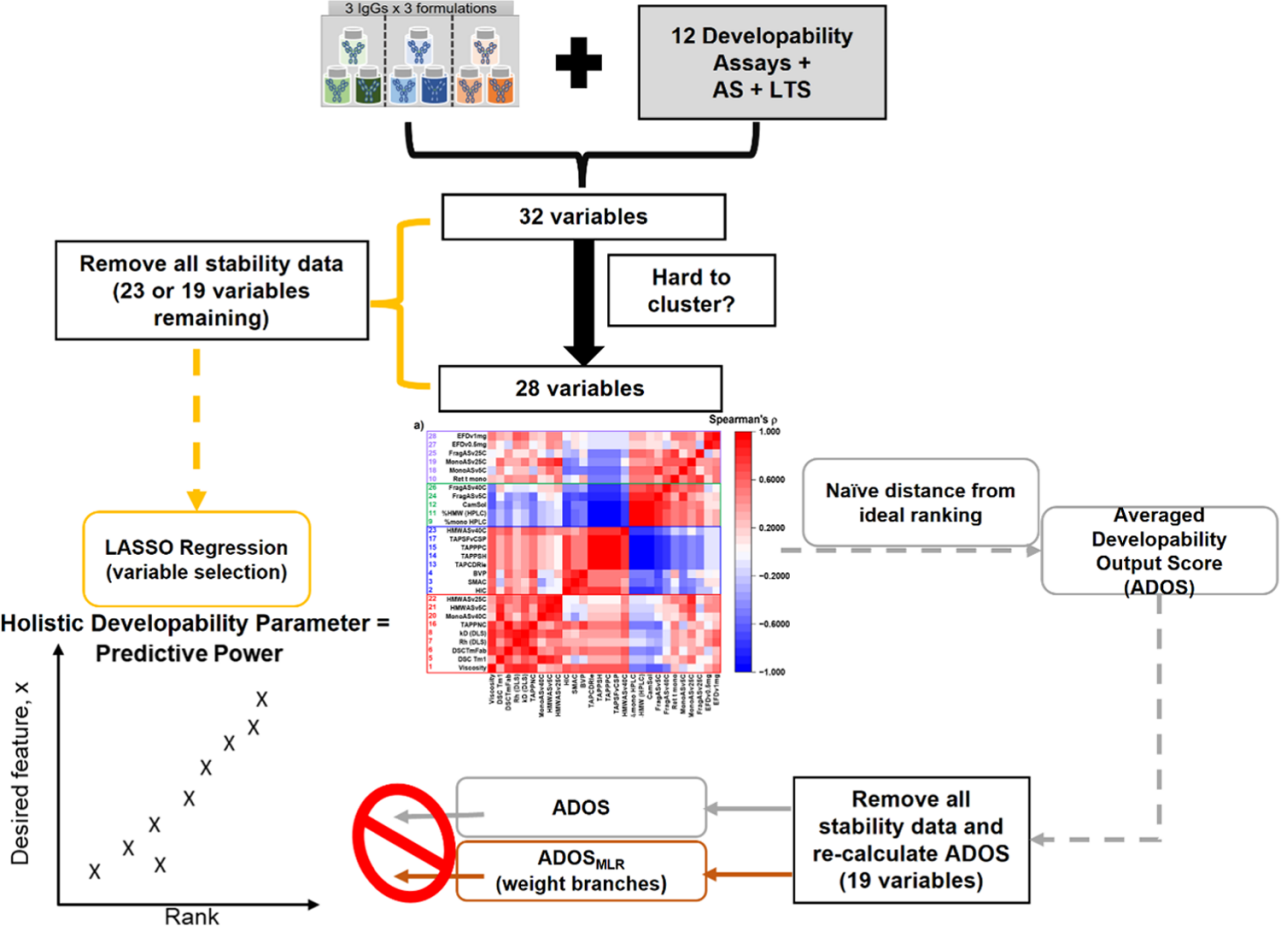
Statistical analysis of the data set yields a Holistic Developability Parameter (HDP). Spearman’s rank and hierarchical clustering identifies the best-clustered set of 28 variables. One can naïvely compute an ADOS from these assay groups to holistically rank formulations (silver arrows). This method is a poor protector of a desired feature (storage stability of 25 °C here). Multiple linear regression (MLR) can be used to optimize ADOS but uses the outputs of all assays (thus we have more variables than data points). An alternative approach uses least-absolute selection and Shrinkage Operator (LASSO) regression to identify which variables contribute to the prediction of the desired feature, as stated above. These key variables make up the HDP.

Each cluster of DAs provides an assessment of distinct biophysical properties (and critical quality attributes), which together determine developability. We thus asked how one could rationally combine DAs to obtain a consensus measure of developability to integrate the often-conflicting results of the DAs employed (Figure 5).

**Figure 6:**
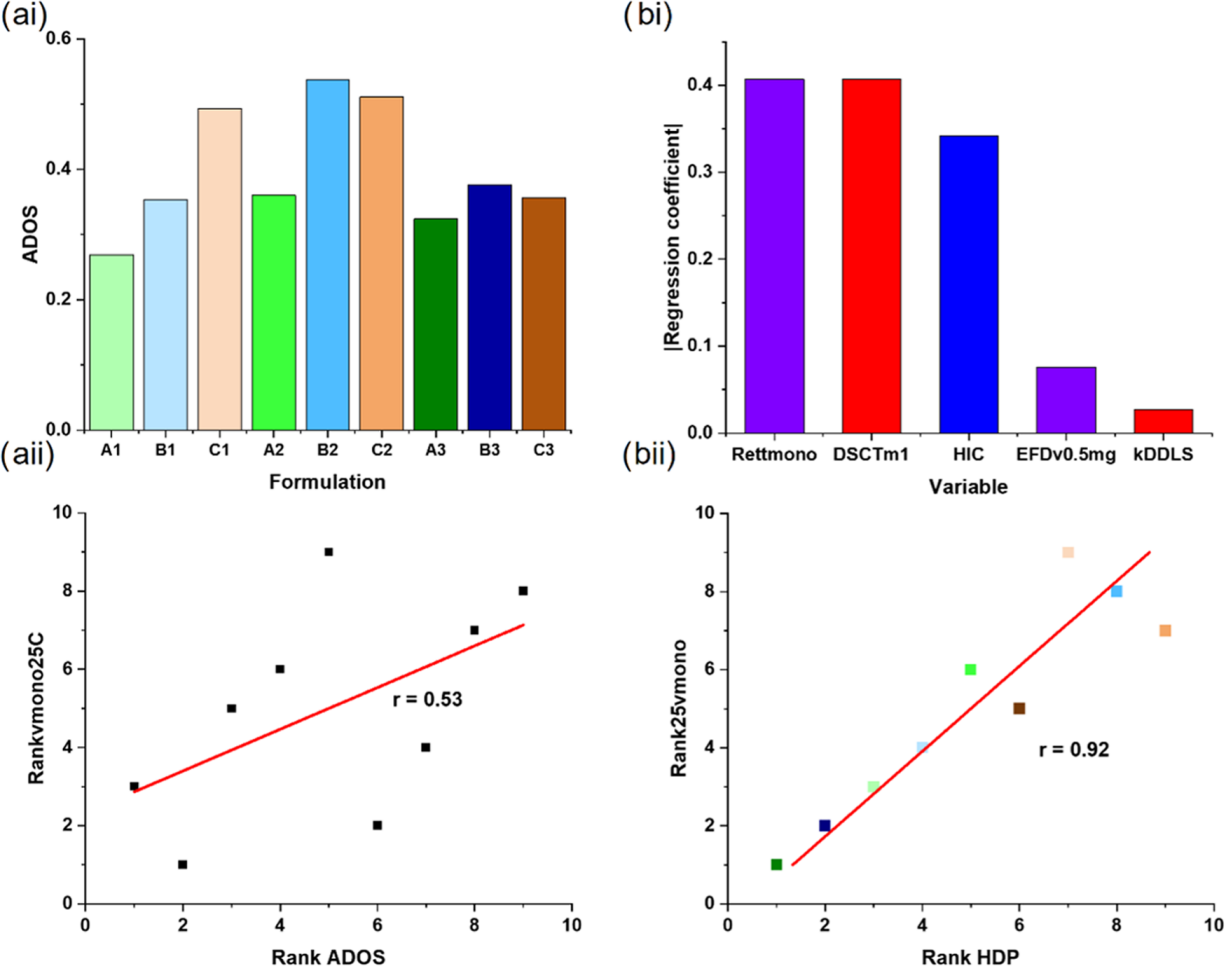
ADOS identifies favorable mAb formulations, while the HDP identifies the “most developable”. (ai) ADOS, derived using the 28 best-clustered variables from Figure 4. Bars are colored by formulation. The ADOS outputs can be put on a rank scale to aid other analyses (Supporting Information) (aii) Rank of observed rate of change in % monomer at 25 °C vs ranked ADOS score. A linear fit to the data shows a modest correlation (*r* = 0.53). LASSO regression of the variable data set excluding accelerated and storage stability (Group V) data identifies the five assay variables (bi) that together yield the HDP which correlates strongly with the ranked observed rate of change in % monomer at 25 °C (*r* = 0.92) (bii). These five diverse assays (abbreviations defined in Tables 3–5) are color-coded in accordance with the dendrogram in Figure 4b.

Inspired by the work of Jain et al.,^4^ where a “distance from ideal” of each test formulation:mAb for each DA was derived, we adapted this analytical framework to generate a parameter to summarize the overall performance of a candidate during developability assessment. For a given variable, each formulation:mAb was ranked on a best (0) to least favorable (1) scale (Methods section). The average score for the assay variables in each branch is then calculated, and the sum of the average values of each branch is calculated. In this model-independent approach, formulation:mAbs with a lower score (herein termed Averaged Developability Output Score, ADOS) are expected to have more quality attributes for developability. This approach first identifies mAb2 as likely to be difficult to develop, as it scores badly in most assay clusters, irrespective of buffer condition (Figure 6ai), and second, identifies Buffer A (histidine-arginine) as the best formulation. One could thus utilize ADOS to consolidate the data from a variety of assays into one, easy-to-interpret metric, reducing the likelihood of one assay variable leading to the outright rejection of a given formulation.

### 3.4 ADOS Cannot Be Used to Assess Storage Stability

While this method yields values that correlate qualitatively with empirical knowledge of the buffers and mAbs used, its ability to identify formulation:mAbs with favorable short-/long-term storage stability was unknown. The prediction of kinetic stability at 5 °C is highly desirable, as this is expensive in terms of both material and time. However, the slow degradation kinetics for the samples studied here precludes this goal for this data set (see the Discussion section). To answer this question, we thus chose the rank order of change in % monomer at 25 °C as the “measured attribute of manufacturability” to be predicted, but we note that other user-defined critical quality attributes could also be used. As all of the accelerated and storage stability data in Group V (obtained at 5, 25, and 40 °C) are expensive in terms of protein required and time to obtain, all Group (V) data were removed from the data set, allowing only rapid “*t* = 0” DAs with low sample requirements to be used to predict storage stability. The remaining 19 variables in the data set were reanalyzed by Spearman’s rank and Hierarchical Clustering, yielding the same four assay clusters identified previously (Figure S22). Plotting the ranked, observed rate of change in % monomer at 25 °C versus the ADOS calculated using the clusters derived from these 19 variables results in a weak correlation (Pearson’s *r* = 0.53, Figure 6aii). As each branch (and assays within branches) may not have equal importance in determining storage stability, Multiple Linear Regression (MLR, Supporting Methods) was employed to weight each branch according to its contribution to this prediction. This made the correlation markedly better (Pearson’s *r* = 0.93, Figure S23), with the caveat that the ADOS_MLR_ is still derived from many different assays, resulting in more degrees of freedom (i.e., variables) than data points.

### 3.5 LASSO Regression Can Be Used to Identify Key Predictor Variables

**Figure 7:**
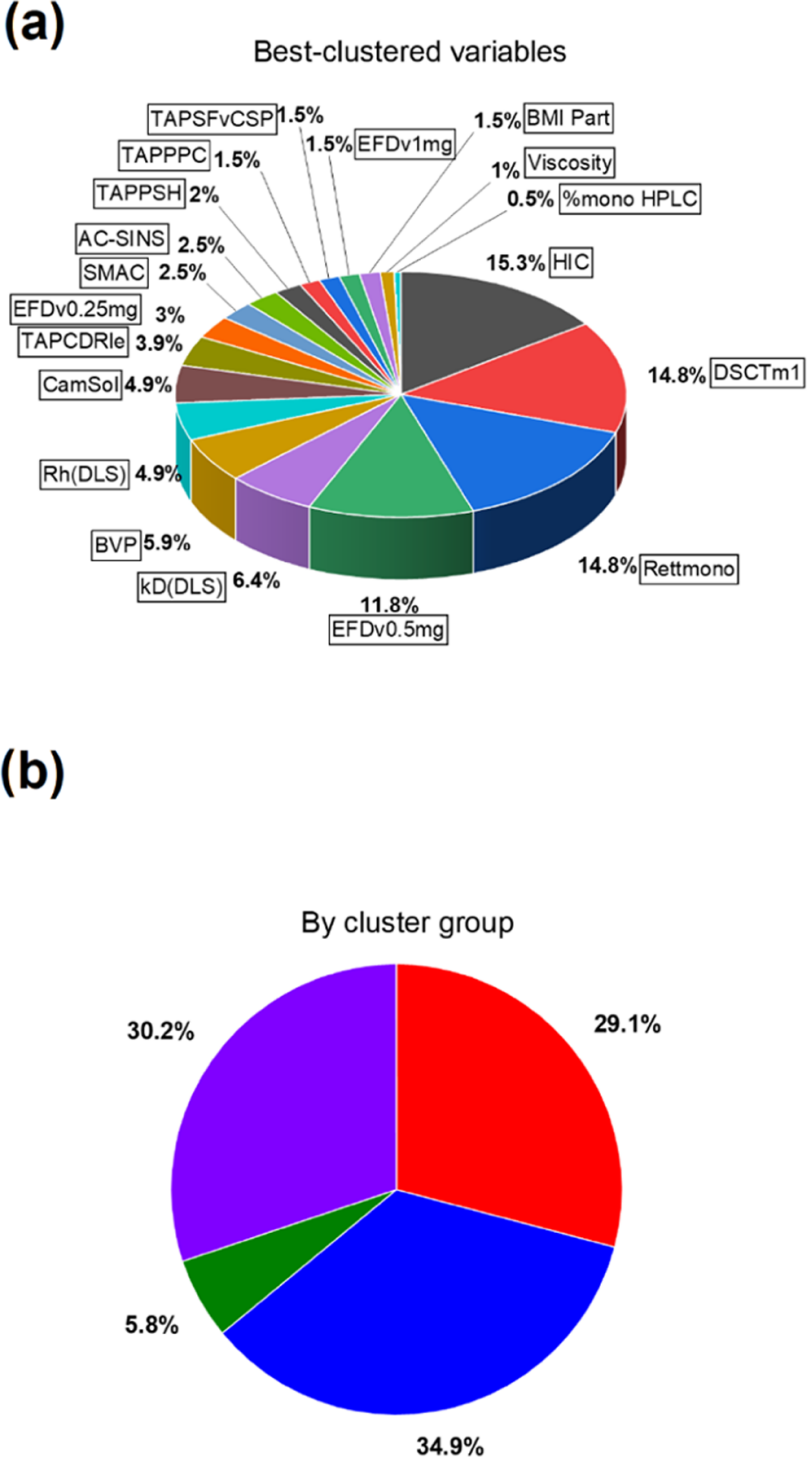
Robustness analysis of the 25 °C storage stability data set and its impact on the LASSO regression. (a) Pie chart showing the selection frequency of the 19 best-clustered variables, which were selected by LASSO regression over the 17 (absolute plus 16 combinations of ranks for A1, B1, A2, and C3) 25 °C data ranks. The five most frequently selected variables are the same as those in Figure 6bi. (b) Pie chart showing the selection frequency of variables in (a) grouped and colored according to the dendrogram in Figure 4. Each LASSO iteration mainly selects variables from the red, blue, and purple assay groups. 5–6 core “*t* = 0” developability assays, spanning an array of physicochemical features, are sufficient to predict 25 °C storage stability.

While the described approaches provide an understanding of the inter-relationship between assays and assay clusters, reducing the number of DAs still requires *ad-hoc* decisions to be made on the data set or a large panel of DAs to be included within the regression against the chosen developability parameter. To obviate this requirement, we adopt a systematic approach that identifies the smallest set of variables to link DAs with the chosen measurable attribute of manufacturability. LASSO regression is a variable selection method that reduces the number of variables to the minimum set that best fit the data, with this method being useful when there are more variables than samples (our data set comprises 19 variables and nine samples, Supporting Methods).^30^ Performing LASSO regression on the data set without Group (V) data reveals that just five assay variables can predict the ranked absolute observed rate of change in % monomer at 25 °C (*r* = 0.92, Figures S24 and 6b). These variables are the first apparent thermal transition in DSC and the *kD* obtained from DLS (Red group, variables 5 and 8, respectively), monomer retention times on HP-SEC (Purple group, variable 10), and HIC columns (Blue group, variable 2) and the observed rate of monomer loss induced by the EFD at 0.5 mg/mL (Purple group, variable 27). Though the regression coefficient for the *kD* is small, removing it from the data set results in no correlation being obtained from LASSO, reinforcing the importance of the *kD* as a developability parameter.^23^ As no information derived from Hierarchical Clustering is used in LASSO regression, we repeated this procedure on the full DA data set (not including the AS or LTS (Group V) data) as the four difficult-to-cluster variables (29 and 32) may still provide important information through their unique insight. LASSO regression once again showed a high correlation with change in % monomer at 25 °C (Pearson’s *r* = 0.95, Figure S24c) but required six variables: the same five as above and one of the difficult-to-cluster variables omitted in previous analyses: the number of particles observed in the formulation:mAbs at *t* = 0 by BMI, (variable 30). Intriguingly, repeating this process to predict the rank order of accelerated stability (% monomer at 40 °C), yielded a lower Pearson’s *r* (0.86) with LASSO regression only selecting the first transition by DSC as an important variable for this (Figure S24c). This together with non-Arrhenius degradation kinetics suggests that monomer loss may occur by different mechanisms at 25 and 40 °C. As noted above, error analysis of the linear regression of the 25 °C degradation rate (Supporting Methods and Figure 3) showed that A3 and B3 have very similar rates of monomer loss at 25 °C and A1, with B1, A2, and C3 displaying varying degrees of significant difference between the observed rates (Figure 3d). To investigate the effect of fitting error on formulation:mAb ranking, the ranks of A3 and B3 were assigned tied first (i.e., most stable), with C2, B2, and C1 (all significantly different to every other formulation:mAb) assigned fixed ranks of sixth, seventh and eighth, respectively. The remaining four formulation:mAbs were systematically reassigned ranks 2–5 using every combination of each of their maximal (high, H) and minimal (low, L) degradation rates, calculated from the fit error, yielding 16 different (2^4^) ranks from LLLL to HHHH. Generally, irrespective of whether the full or focused variable data set was used for LASSO regression, the predictive power (Figures S25 and S26) and identified keystone variables (Figure 7) are preserved. As a median of six keystone variables, which probe diverse physicochemical features of the molecules, are selected from these analyses (Figure 7), this suggests our approach, which generates a Holistic Developability Parameter (HDP) could work as a general strategy for mAb developability.

## 4 Discussion

An ever-expanding toolkit of developability assays has been established by the field to interrogate various physicochemical features of antibodies, with a view to identifying lead candidates with favorable drug-like properties.^2,4,45^ Studies have subjected panels of IgG antibodies^4,7,12,20,46−48^ and other modalities^19,49^ to various established^9,50,51^ and novel DAs^13,17^ and analyzed the resulting data sets by a variety of statistical methods including Pearson’s^12,19^ and Spearman’s correlation.^4,16,49^ The majority of these studies have investigated the relationship (and potential redundancies) between different DAs including a wide array of well-used assays^4^ or the relationship between these established DAs and novel assays that report on hydrodynamic and interfacial stability^12,13,16^ or in silico-derived parameters.^47^ Other groups have examined the ability of DAs to predict the behavior of proteins during downstream processing.^20,23^ Interestingly, and in agreement with our results, both of these latter studies identified parameters that measure self-association, such as *kD*, to be the strongest predictors. Despite these successes, a framework for the integration of the diverse outputs of DAs was lacking. This challenge is nontrivial, based on the array of mAb sequences available,^45^ the orthogonal set of assays one can use to interrogate these molecules^2,47^ and the impact that different formulation components (namely, buffers, cosolutes, and excipients) can have on the above.^31^

We subjected a panel of three mAbs in three different formulations to an array of DAs and measured their accelerated and long-term stability over a three to 18-month period. Spearman’s rank was chosen to assess correlations between the resulting assay variables, as this avoids the potential bias from assuming linear correlations between different variables and reduces the influence of measurement noise on the analysis.^52^ By employing Hierarchical Clustering on the Spearman correlation coefficients, we were able to identify DAs that group readily into families (e.g., HIC and BVP), as well as four DAs that were hard to cluster (AC-SINS, BMI, initial levels of fragmentation by HP-SEC and the observed rate of EFD-induced monomer loss at 0.25 mg/mL). For AC-SINS, poor clustering may be due to the atypical blue shifts observed in Buffer B (Figure S4, possibly caused by a change in the stability of the nanoparticles themselves).^53^ For the EFD data, we postulate that surface-dominated aggregation occurs at low protein concentrations with a second bulk aggregation pathway occurring at higher concentrations (Figure S11).^28^ It is important to note that “difficult to cluster” may instead indicate that these assays probe unique features of the molecules as shown when the outputs of the EFD assay applied to subset of the “Jain” panel of mAbs were compared to the other DAs,^16^ as well as outputs derived from charge-stabilized self-interaction nanoparticle spectroscopy and poly specificity particle assays performed on a set of 80 clinical-stage sequences.^46^

We utilized 12 DAs at *t* = 0, as well as performing a stability study at three temperatures (5, 25, and 40 °C) for 18, 6, and 3 months, respectively. While we did monitor the change in the macroscopic properties of the samples using visual inspection standards,^54^ the noncontinuous nature of the data generated precluded their inclusion in our final workflow. Furthermore, the particulate matter was tracked over the course of the stability study using background membrane imaging. Many 40 °C samples exceeded the recently derived^55^ measurement limits for the technique after just 1 month (data not shown); hence, only the *t* = 0 data were used in the final data set. Transforming the outputs of the 28 best-clustered variables from these assays to a single scale allowed us to understand how best to utilize these data. First, by assuming all assays are equally important, we condensed the complex and sometimes conflicting DA outputs to a single measure of biophysical behavior (the ADOS), in a similar fashion to the “distance from ideal” measurement derived by Jain et al. 2017, though other normalization methods have been developed recently.^51^ The distance from ideal values was used by Jain et al. to then cluster the 137 IgG molecules in their study into groups of well-behaved (i.e., developable) sequences, as well as those with less favorable properties, without explicitly ranking these from best to worst or investigating the consequences of “nondevelopability” on kinetic stability, for example. Rattray and colleagues condensed their DAs using a normalization method, summing these scores but attributing no weighting to, e.g., different families of assays, as hierarchical clustering was not employed on their ranked data. They showed that a lower normalized score correlated with reduced viscosity for a panel of high-concentration mAbs.^51^

The ADOS method identified the arginine-containing Buffer A as the formulation that yields the best-behaved molecules (in terms of biophysical properties), but it is a poor predictor of the exemplar used to test our manufacturability prediction, that of kinetic stability at 25 °C. This is probably because inherent within the ADOS methodology is the assumption that all assays within a branch and all branches are equally important. Using a similar approach Wolfgang Freiss and colleagues showed that a modest correlation was observed between aggregation after six months at 4 and 25 °C (the data for both temperatures and all formulations was averaged) and a “Stability Risk Score By High Analytical Effort” derived from 16 variables.^19^ Similarly to the ADOS approach, this work also suggested that the formulation largely determined the output score.^19^ By essentially removing unimportant variables (in terms of predictive power), LASSO regression is a powerful method to identify the subset of assay variables and optimize the weightings necessary to predict kinetic stability at 25 °C. In contrast to the multiple studies to delineate the relationship between DAs, studies investigating the relationship between DAs and kinetic stability at 5, 25, and 40 °C are less common. Goldberg et al. assessed DAs such as Tm,app, aggregation onset temperature, and 40 °C aggregation and monomer-loss rates for a panel of mAbs in different formulations. They found the strength of the correlation was dependent on the formulation condition and that the correlation with 40 and 4 °C data was poor.^38^ Others have also shown that it is difficult to correlate the behavior of different DAs with real-time stability, based on the molecules and formulations in question and the temperature dependence of their underlying degradation mechanism.^19,21,49^

Comparing the outputs from the independent approaches of hierarchical clustering and LASSO regression shows that HDP integrates variables from different branches of the “family tree” of clustered assays, which report on a range of biophysical properties: thermal and colloidal stability (Tm1 by DSC and *kD* by DLS), stickiness (HIC and SEC retention time), and sensitivity to interfacial and hydrodynamic stresses (EFD). The emergence of colloidal stability accords with a wealth of previous studies that link this property to downstream processing and solution behavior.^20,23,51^ The non-Arrhenius kinetics exhibited by our formulation:mAbs and reported in other studies,^36−38^ prevents the use of recently established kinetic models to directly predict long-term stability from our accelerated stability data.^39−41,56,57^ and also suggests that aggregation (or any other process that drives the monomer loss used as a metric of manufacturability used here) may be driven by transient partial unfolding of the native state. This accords with monomer loss increasing with decreased Tm1, increased HIC and SEC retention time, and increased sensitivity to interfacial and hydrodynamic stresses. Given this broad sampling of biophysical characteristics and its strong correlation with the ranked stability data obtained at 25 °C, we have termed this the Holistic Developability Parameter or HDP. The fact that our developability framework, which utilizes many diverse assays, was able to describe this correlation suggests that monomer loss occurs by distinct pathways that do not simply involve global unfolding and that the generality of our approach removes the need for the user to have a detailed knowledge of the aggregation mechanism underpinning a given protein’s degradation pathway.^42^ Similarly, as the HDP is determined by diverse assays (i.e., four of the five families of related assays, Figure 6), these assays may be sufficient to broadly define the biophysical and chemical behavior of proteins. Consequently, the same core assays may be sufficient to predict a variety of critical quality attributes but using an HDP comprising different weightings for each assay. The prediction of 5 °C long-term storage stability using LASSO was precluded by the stability of the formulation:mAbs in our test set at this temperature. This may be because the mAbs used in our study (and others^21^) had all reached later stages of development. Accordingly, we suggest that the method we describe is employed as a rapid screen during candidate selection to ensure identification of mAbs with suitable long-term stability after sequence-based features which are linked to inherently poor developability, e.g., charge and hydrophobicity,^58^ or low chemical stability are removed using online tools such as LAP.^59^ Here, we have focused on predicting kinetic stability at 25 °C, as this parameter is onerous to measure in terms of time (six months) and material (>200 mg per molecule). We reiterate that our approach provides a general framework to define the key assays that predict any measure of manufacturability of the user’s interest, provided a test data set of the outputs of a variety of DAs, together with the parameter of interest to be predicted, has been measured for a panel of mAbs. Here, in contrast to more complex machine-learning methods (which may nonetheless employ LASSO regression), we have used a relatively small data set and LASSO regression to generate a simple and sparse predictive model containing five or six key variables, using assays that consume milligram quantities of material and take less than a day to complete. Furthermore, these variables stem from different branches of the “family tree” of assays, thus encompassing a range of biophysical features of each formulation:mAb. Of course, more molecules, covering number, sequence, topology, protein concentration, and formulation diversity, will be needed to test this further in the future, with some data sets already emerging to this aim,^49^ providing the groundwork needed to test our general framework’s broad applicability.

## 5 Conclusions

Herein, we subjected nine different formulation:mAbs to an array of diverse lab- and computer-based developability assays, alongside the rate of relative monomer loss at 5, 25, and 40 °C to obtain a test data set with which to develop a rational framework for DA selection. Through adopting a robust statistical approach, we demonstrate that it is possible to identify a minimal set of DAs capable of predicting a specific critical quality attribute of the development pipeline. Combining these variables using the LASSO approach yields a quantifiable HDP by which candidates can be ranked by a user-determined measure of manufacturability irrespective of often-conflicting results from multiple, separate DAs. Here we demonstrate the approach by using day zero DAs to predict the storage stability at 25 °C, since the latter is expensive (in terms of both time and material) yet essential within the regulatory framework. The streamlining of development in this way supports intensification within the drug pipeline, reducing costs and increasing sustainability.

## Supporting Information Available

Further information on the developability assays; data distributions for each assay variable; raw data for the AS and EFD studies; and statistical analysis; main analyzed data, including the ranked data, are available via the University of Leeds repository (10.5518/1470), and other data are available from the corresponding authors upon reasonable request (PDF)

## Notes

I.T., J.C.S., K.D., N.B., P.W.A.D., C.L., and N.J.D. are all employees of AstraZeneca PLC. M.G.B. was an employee of AstraZeneca PLC at the inception of the study.
