# Supplementary material for: Rationalizing mAb Candidate Screening Using a Single Holistic Developability Parameter
Source: Mol Pharm. 2024 Dec 16;22(1):181–95. doi: 10.1021/acs.molpharmaceut.4c00829 (PMC11707744; doi:10.1021/acs.molpharmaceut.4c00829)
Supplement: Supplementary file 1 — mp4c00829_si_001.pdf [file mp4c00829_si_001.pdf]

Supporting information for "**Rationalising mAb candidate screening using a single holistic developability parameter**"

Leon F. Willis,[a,b] Isabelle Trayton,[c] Janet C. Saunders,[c] Maria G. Brùque,[c#] William Davis Birch,[d] David R. Westhead,[a] Katie Day,[c] Nicholas J. Bond,[c] Paul W.A. Devine,[c] Christopher Lloyd,[c] Nikil Kapur,[d] Sheena E. Radford,[a,b] Nicholas J. Darton[c*] and David J. Brockwell[a,b*]

Supplementary Methods

Measurement of osmolality and pH of final (50 mg/mL) AS and LTS samples

Following dialysis (methods) and concentration determination, the pH of the final 50 mg/mL formulation:mAb samples were determined using a Mettler Tolledo pH meter. To determine the osmolality of the final samples, 3 × 20 µL of each sample was measured using an OsmoPro osmometer. The final values are reported in Table S1.

Rheology of surfactant-free formulations

The nine formulation:mAb samples were concentrated using centrifugal filters (30,000 MWCO, Merck Millipore) and the protein concentration determined using a Trinean spectrophotometer, with three 3 µL replicates measured at 280 nm and averaged. All proteins were then diluted to the lowest 'maximum' concentration = 131 mg/mL. The dynamic viscosity of the respective formulation:mAb samples was then determined using an Anton Paar Physica MCR301 cone and plate rheometer. 150 µL of each sample was loaded onto the plate, then a constant shear rate of 1000 s^{-1} applied over 30 min. From the average measured shear stress, the viscosity is determined. These measurements were performed prior to addition of PS80 (see main text).

Hydrophobic Interaction Chromatography (HIC)

HIC was performed as described in Jain et al.[1] Each formulation:mAb (50 mg/mL) was thawed from frozen stocks in Leeds, UK and serially diluted with the appropriate formulation buffers to 1 mg/mL. After 1:1 dilution with running buffer A (0.1 M sodium phosphate (Sigma) and 1.8 M ammonium sulfate (ThermoScientific), pH 6.5) the vials (300 µL conical insert vials, VWR) were crimp-sealed with PTFE seals (ThermoScientific). 5 µg of each sample was loaded onto a Butyl HIC column (Sepax) with a Shimadzu Nexera HPLC system, eluting with a 0–100% gradient of Buffer B (0.1 M sodium phosphate, pH 6.5) at 1 mL/min over 25 mins. Sample elution was monitored at 280 nm with a PDA detector. The retention time of the peak was extracted from the chromatogram in the LabSolutions software supplied with the instrument.

Stand-up Monolayer Adsorption Chromatography (SMAC)

1mg /mL solutions of each formulation:mAb were prepared and loaded into HPLC vials as described above for HIC. 2 µg of sample was injected onto a Zenix column (Sepax) mounted

on a Shimadzu Nexera system, eluting isocratically with 0.15 M sodium phosphate pH 7.0, at a flow rate of 0.35 mL/min. Proteins were detected at 280 nm and retention times were extracted from the chromatograms of each sample.

Baculovirus Particle Enzyme-linked Immunosorbent Assay (BVP-ELISA)
5×10^{10} particles of baculovirus were adsorbed onto wells of a Nunc Maxisorp plate in 50 mM sodium carbonate buffer pH 9.6. A control plate containing buffer only was also prepared. Plates were incubated overnight at 4 °C. After removal of excess unbound baculovirus, plates were blocked with 50 µL of blocking buffer (PBS containing 0.5% (w/v) BSA, Sigma A9576) for 1 hr at room temperature. Plates were washed three times with PBS before addition of 50 µL of 10 nM or 100 nM antibody diluted in PBS, buffer A, buffer B or buffer C containing 0.5% (w/v) BSA. Buffer-only control wells were also prepared. After a 1 hr incubation, the unbound antibody was removed by washing the plates three times in PBS. The extent of binding was determined by addition of 50 µL anti-human IgG antibody conjugated to the horseradish peroxidase (HRP) enzyme (Sigma, A0170) diluted 1:5000 in blocking buffer to all wells. Plates were incubated for 1 hr at room temperature, washed three times in PBS and 50 µL of TMB substrate (KPL 53-00-03) added to each well. Reactions were stopped after 15 min by addition of 50 µL 0.5 M H_2SO_4 and absorbance measured at 450 nm. BV scores were calculated for the BV-coated and non-BV coated plates by dividing the absorbance at 450 nm for each antibody by the buffer-only control wells.

Affinity Capture Self-Interaction Nanoparticle Spectroscopy (AC-SINS)
AC-SINS was performed as described in [1,2], with some modifications. Polyclonal goat anti-human IgG Fc antibodies ("capture"' Jackson ImmunoResearch, 109-005-098) and goat IgG ("non-capture"; Jackson ImmunoResearch, 005-000-003) were buffer exchanged into 20 mM sodium acetate (pH 4.3) using PD-10 columns (GE Healthcare). Samples were concentrated (100 kDa centrifugal filters, Merck Millipore) and normalised to 0.4 mg/mL. 900 µL citrate-stabilized 20 nm gold nanoparticles (Innova Biosciences, 3201-0100) was mixed with 800 µL "capture" and 200 µL "non-capture" antibodies to make an 80% capture capacity nanoparticle solution. After incubation for 1 hr at room temperature, a final concentration of 0.1 µM poly(ethylene glycol) methyl ether thiol (Sigma-Aldrich) was added to block empty sites on the nanoparticles. The solution was mixed by inversion and incubated at room-temperature for 1 hr. The nanoparticles were pelleted at 13,000 rpm (6 min) in siliconized Eppendorf tubes (VWR, 22179-004) and resuspended in 800 µL of the supernatant.

In a 96-well polypropylene plate (Agilent Technologies), 10 µL of the nanoparticle solution was incubated with either 100 µL 50 µg/mL test antibody solution in PBS or buffer A, buffer B or buffer C, or with 100 µL of buffer-only, for 2 hr at room temperature. 50 µL of each solution was transferred to two wells of a transparent polystyrene 384-well plate (Thermo Scientific) followed by a 1 min centrifugation to bring menisci to an equal level. The absorbance was read from 510 to 570 nm (2 nm increments) using a PHERAstar FSX plate reader. The MARS data analysis software was used to determine the wavelength that resulted in the maximum absorbance for each sample. To reduce error from noise, curves were smoothed before the calculation using the software. The difference in wavelength of maximum absorbance between the buffer-only control and the test samples were calculated to determine

the shift in wavelength of maximal absorbance. A shift of >10 nm identified the test antibody as being at risk of self-association.

Differential Scanning Calorimetry (DSC)

mAb thermal stability in each formulation at t =0 was characterised using a Malvern Microcal VP-Capillary Differential Scanning Calorimeter. All samples were diluted ten-fold to 5 mg/mL. 200 µL of each sample was loaded into a deep well plate (Malvern Panalytical), alongside Milli-Q water and buffer blanks. Scanning was performed from 25 to 110 °C at a scan rate of 1.6 °C per minute. Lyophilised Hen Egg White Lysozyme (Sigma) was reconstituted in Milli-Q water to a concentration of 3 mg/mL and used as an internal control[3] Data were analysed using the "Origin for DSC" plug-in for OriginPro, fitting the data to a non two-state model.

Dynamic Light Scattering (DLS)

Each formulation:mAb was prepared at 2-, 4-, 6-, 8-, 12-, 16- and 20 mg/mL, then filtered through low dead volume 0.22 µm syringe filters (Merck Millipore). Three 30 µL samples of each formulation:mAb was then loaded into a 384-well plate in a laminar flow hood (Corning). After spinning in a plate centrifuge (1 min, Eppendorf), dynamic light scattering was measured at 25 °C over 3 hr, using a Wyatt DynaPro instrument. The data were analysed using the Dynamics software supplied with the instrument. The average hydrodynamic radius was calculated in Microsoft Excel. For each formulation, the diffusion interaction parameter (k_D) was extracted from a linear fit to a plot of diffusion co-efficient versus protein concentration using Equation S1

$$D=D_0*(1 + k_D*c)$$

Equation S1. where D = Diffusion coefficient, D_0 = diffusion coefficient close to zero concentration, c = concentration (in g/mL) and k_D is the diffusion interaction parameter (mL/g). Protein concentration of each diluted sample was determined post-experiment using a Trinean Dropsense spectrophotometer (details below).

Background Membrane Imaging (BMI)

BMI was performed using a Horizon instrument. Initially, the hydrophobic 96-well sample plate (HaloLabs) was imaged in the software. Then, 25 µL of sample (three wells per sample) was pipetted into each well in a laminar flow hood. The samples were then filtered through a vacuum manifold, then washed with 25 µL of water per well. Following a final vacuum filter step, the plate was blotted dry, then the plate imaged in the Horizon instrument. The software subtracts the earlier background image to increase the contrast between the background and the particles. The data were extracted, then analysed with Microsoft Excel.

Therapeutic Antibody Profiler (TAP)

The variable heavy (V_H) and light (V_L) sequences of mAbs 1–3 were inputted into the TAP webserver (https://opig.stats.ox.ac.uk/webapps/newsabdab/sabpred/tap,[4]), as described previously.[5] Briefly, the webserver builds a homology model of the input protein using ABodyBuilder (version = ABodyBuilderML at the time of analysis), then derives five different *in-silico* metrics from the model: Total CDR length (TAPCDRle), Patches of Surface Hydrophobicity (TAPPSH), Patches of Positive Charge (TAPPPC), Patches of Negative Charge (TAPPNC) and Structural Fv Charge Symmetry Parameter (TAPSFvCSP). These

metrics are compared against an evolving database of approved clinical antibody sequences, with our analysis performed on 27th June 2022.

CamSol algorithm

As described previously,[5] the homology models output from TAP (see above) were input into the CamSol webserver ([6] http://www-vendruscolo.ch.cam.ac.uk/camsolmethod.html). Briefly, the models are annotated with patches thought to promote or disfavour solubility. The structure-corrected CamSol scores were output and used for subsequent analysis. The output model structures were visualised in UCSF Chimera (version 1.17).[7]

Soluble protein concentration determination of DLS, accelerated (AS) and long-term stability (LTS) samples

As a quality control procedure, the concentration of soluble protein in the DLS, AS and LTS samples was measured using a Trinean spectrophotometer. 3 µL of sample was measured at 280 nm, together with each respective formulation buffer as a blank. The protein concentration was determined using each protein's molar absorption extinction coefficient. The protein concentration for each mAb in the AS and LTS samples are shown in Figure S13, with the average across the study shown in Table S2.

Multiple Linear Regression

To investigate whether the Averaged Developability Output Score (ADOS) correlated with storage stability, variables which pertained to accelerated and storage stability at 5°C, 25°C and 40°C (the observed rates of change in % monomer, % HMW species and fragmentation at each respective temperature) were removed from the dataset, leaving 19 variables. Hierarchical clustering places the remaining variables in the same four branch family tree (Figure S22).

After re-calculating the ADOS with these variables, then re-ranking the ADOS values, the linear correlation between this rank and the ranked observed rate of change in % monomer at 25 °C was calculated using OriginPro. The latter metric was chosen as statistically significant changes in monomer concentration were observed at this temperature. To attempt to optimise the fit, multiple linear regression was performed in Origin Pro, using each branch score as the input. The β- coefficients (the weighting function) for each branch with the 25 °C observed change in % monomer rate were: Group 1, red, 7.44562; Group 2, blue, -6.61587; Group 3, green, -2.2698; Group 4, purple, 1.90604). (Figure S23). The $Grp\bar{x}$ variables were multiplied by their respective β- coefficients and the new $ADOS_{MLR}$ computed and ranked (with the most negative value being the "lowest" and therefore best molecule).

Supplementary Data

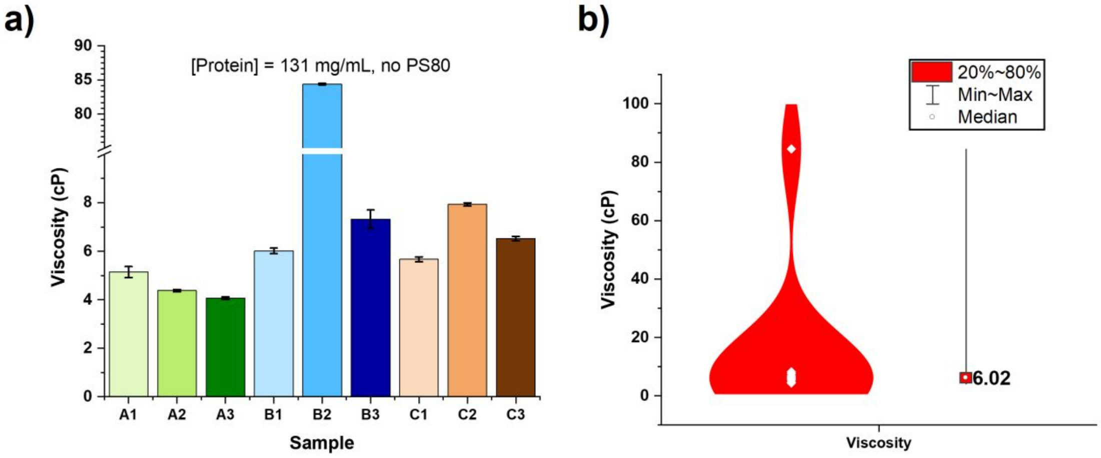

Figure S1. Characterisation of the viscosity of the surfactant-free formulations at t=0. a) Average viscosity of the formulation:mAbs (error bars = s.d. over 30 measured points). b) Violin plot (left) and box and whisker diagram (right) showing the distribution of the viscosity data. The box boundaries are the 20% and 80% percentiles. The median value is shown in bold. The bar spans the range of the data and is coloured in accord with the grouping in the Hierarchical Clustering dendrogram (Figure 4b).

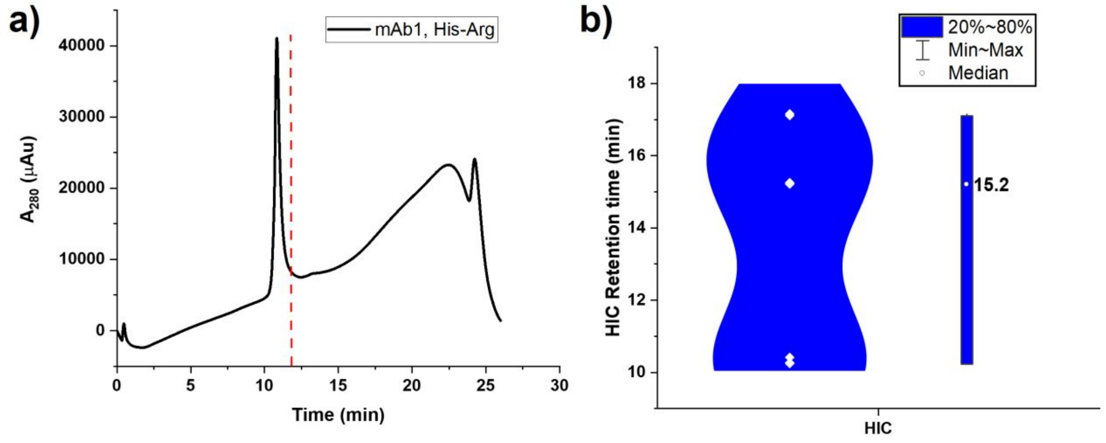

Figure S2. Characterisation of the hydrophobicity of the formulation:mAb panel at t=0 by Hydrophobic Interaction Chromatography (HIC). a) Representative chromatogram for formulation:mAb A1. For a frame of reference, the red dashed line indicates the threshold value, above which a mAb was deemed unfavourable in Jain et al 2017.[1] b) Violin plot (left) and box and whisker diagram (right) showing the distribution of the retention time data. The box boundaries are the 20% and 80% percentiles. The median value is shown in bold. The bar spans the range of the data. The diagram is coloured in accord with the grouping in the Hierarchical Clustering dendrogram (Figure 4b).

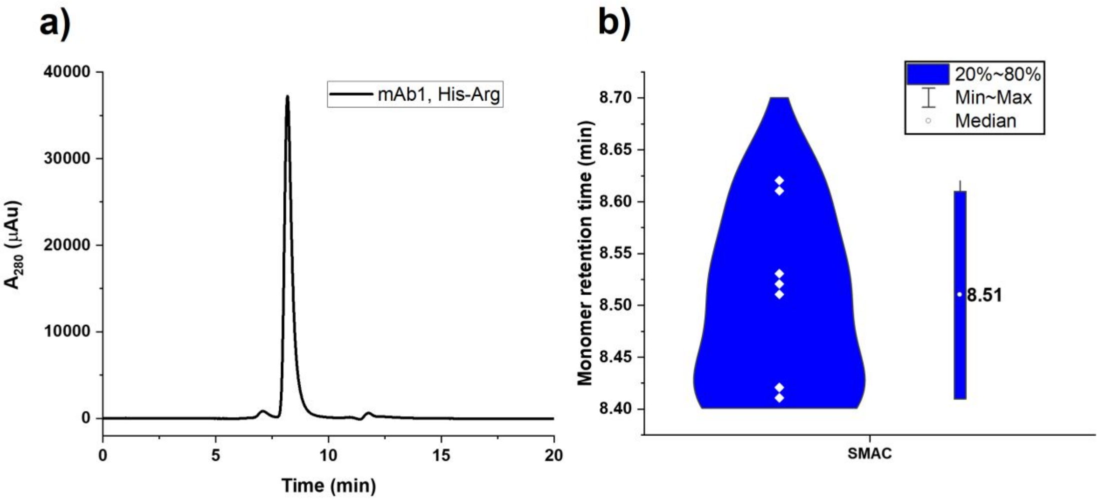

Figure S3. Characterisation of the formulation:mAb panel at t=0 by Stand-up Monolayer Adsorption Chromatography (SMAC). a) Representative chromatogram for formulation:mAb A1. The major peak represents the monomer. b) Violin plot (left) and box and whisker diagram (right) showing the distribution of the retention time data. The box boundaries are the 20% and 80% percentiles. The median value is shown in bold. The bar spans the range of the data. The diagram is coloured in accord with the grouping in the Hierarchical Clustering dendrogram (Figure 4b).

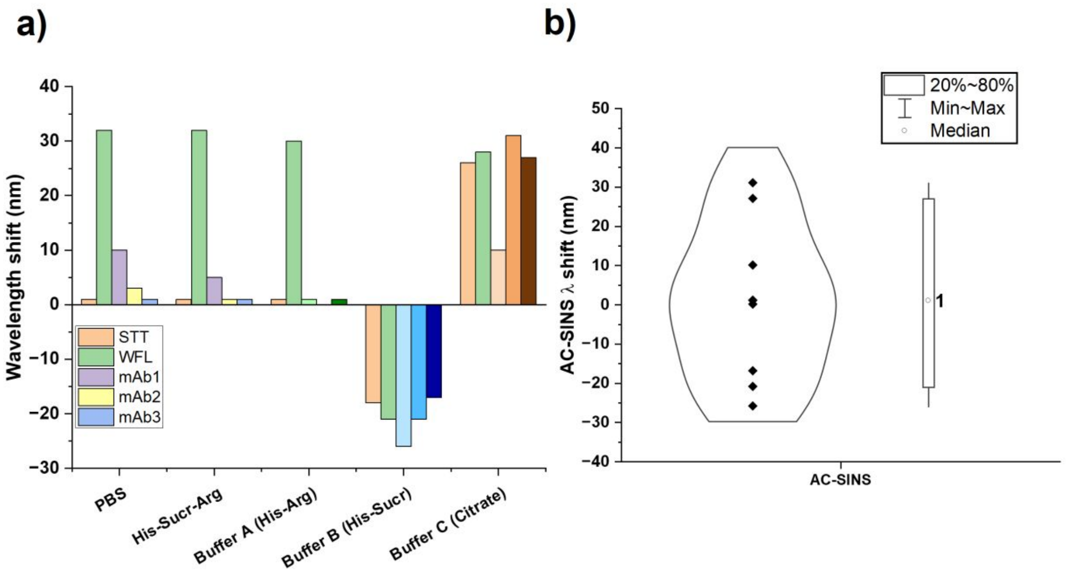

Figure S4. Characterisation of the formulation:mAb panel at t=0 by Affinity Capture-Self Interaction Nanoparticle Spectroscopy (AC-SINS). a) Plasmon wavelength shift (Δλ) of functionalised gold nanoparticles in the presence of each antibody formulation. STT is used as internal standard of a well-behaved mAb, with +ve control and WFL (refer to Dobson et al. 2016)[8] being poorly behaved mAbs. b) Violin plot (left) and box and whisker diagram (right) showing the distribution of wavelength shift data. The box boundaries are the 20% and 80% percentiles. The median value is shown in bold. The bar spans the range of the data. The diagram is colourless, due to its subsequent exclusion from the final suite of assay variables.

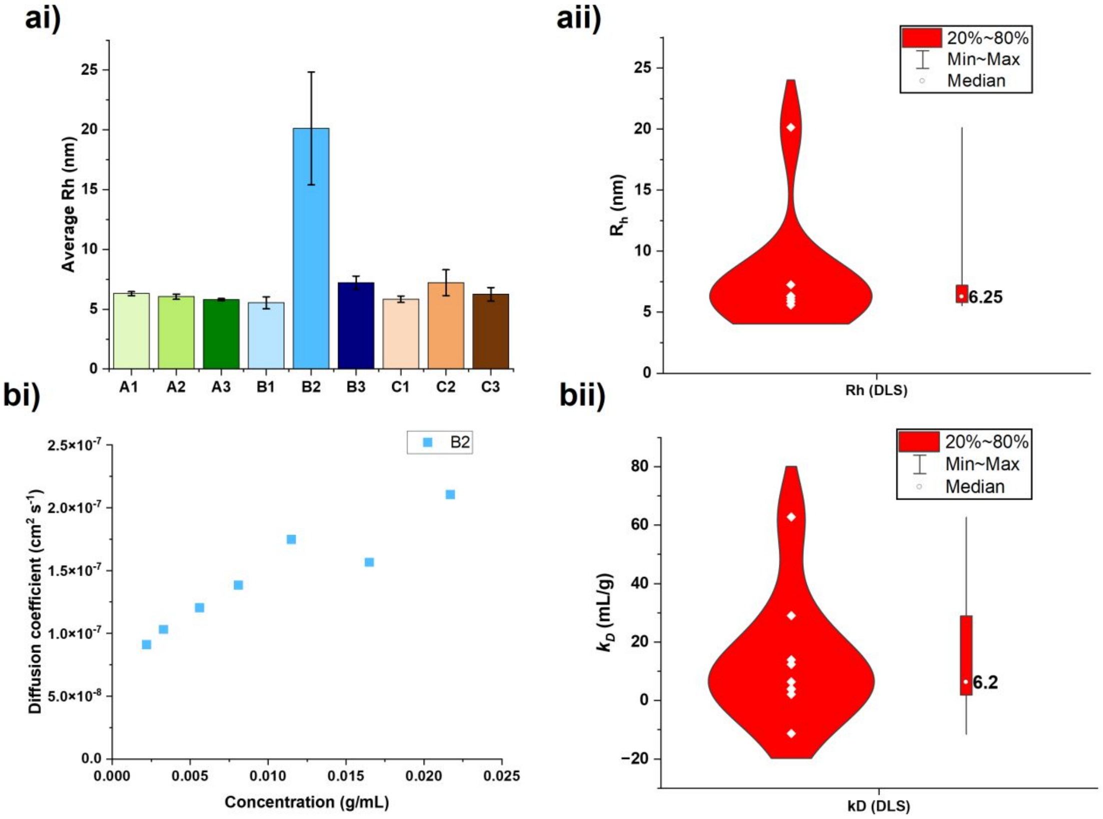

Figure S5. Characterisation of the formulation:mAb panel at t=0 by Dynamic Light Scattering (DLS). ai) Average hydrodynamic radius (R_h) of the mAbs in each formulation, across the concentration range studied. aii) Violin plot (left) and box and whisker diagram (right) showing the distribution of the R_h data. bi) Plot of diffusion coefficient against concentration of formulation:mAb B2, obtained from the DLS analysis. bii) Violin plot (left) and box and whisker diagram (right) showing the distribution of the k_D values extracted from fitting Equation S1 to the DLS data (Supplementary Methods). In aii and bii, the box boundaries are the 20% and 80% percentiles. The median values are shown in bold. The bars span the range of the data. The diagram is coloured in accord with the grouping in the Hierarchical Clustering dendrogram (Figure 4b).

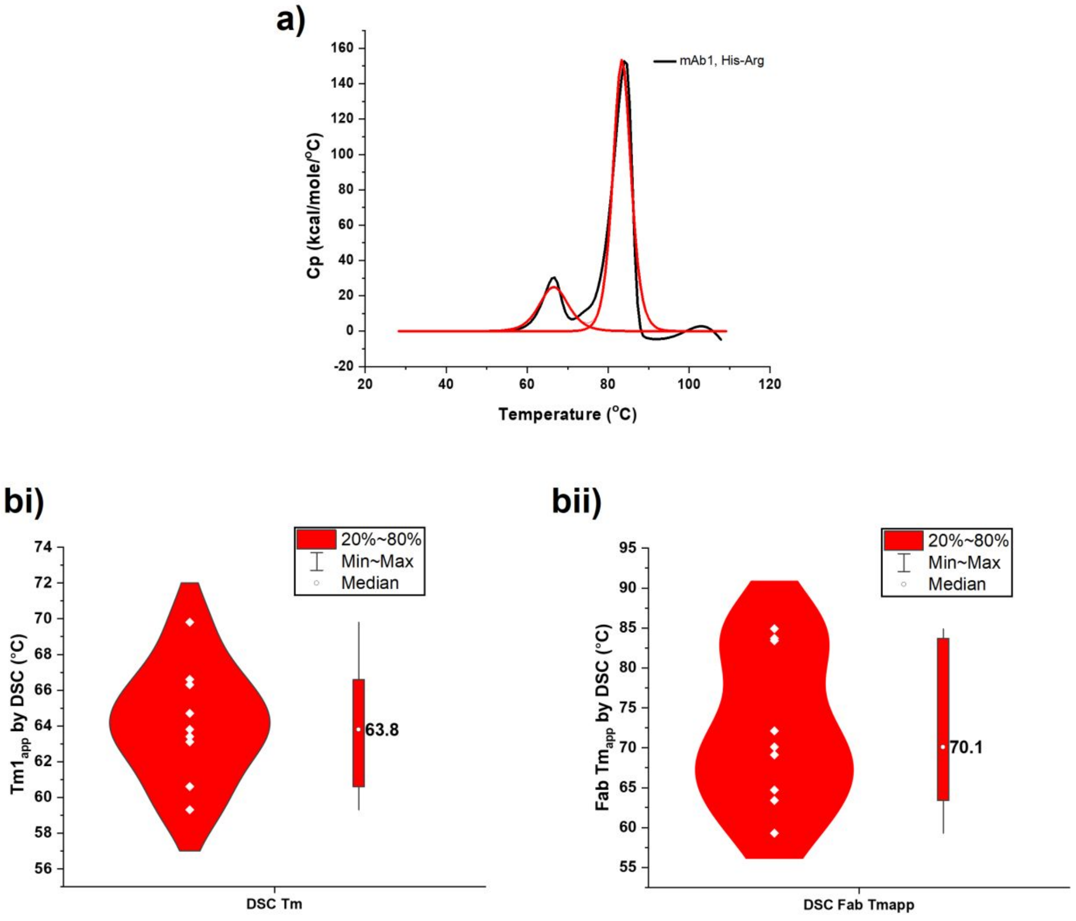

Figure S6. Characterisation of the formulation:mAb panel at t=0 by Differential Scanning Calorimetry (DSC). a) Representative DSC thermogram for formulation:mAb A1. The red lines represent the fit of the data (black) to a non two-state model (Supplementary Methods). bi) Violin plot (left) and box and whisker diagram (right) showing the distribution of the apparent Tm data for the 1st transition ($Tm1_{app}$). bii) Violin plot (left) and box and whisker diagram (right) showing the distribution of the Tm_{app} for the Fab (corresponding to the major peak in the thermogram of each respective sample). In bi and bii, the box boundaries are the 20% and 80% percentiles. The median values are shown in bold. The bars span the range of the data. The diagram is coloured in accord with the grouping in the Hierarchical Clustering dendrogram (Figure 4b).

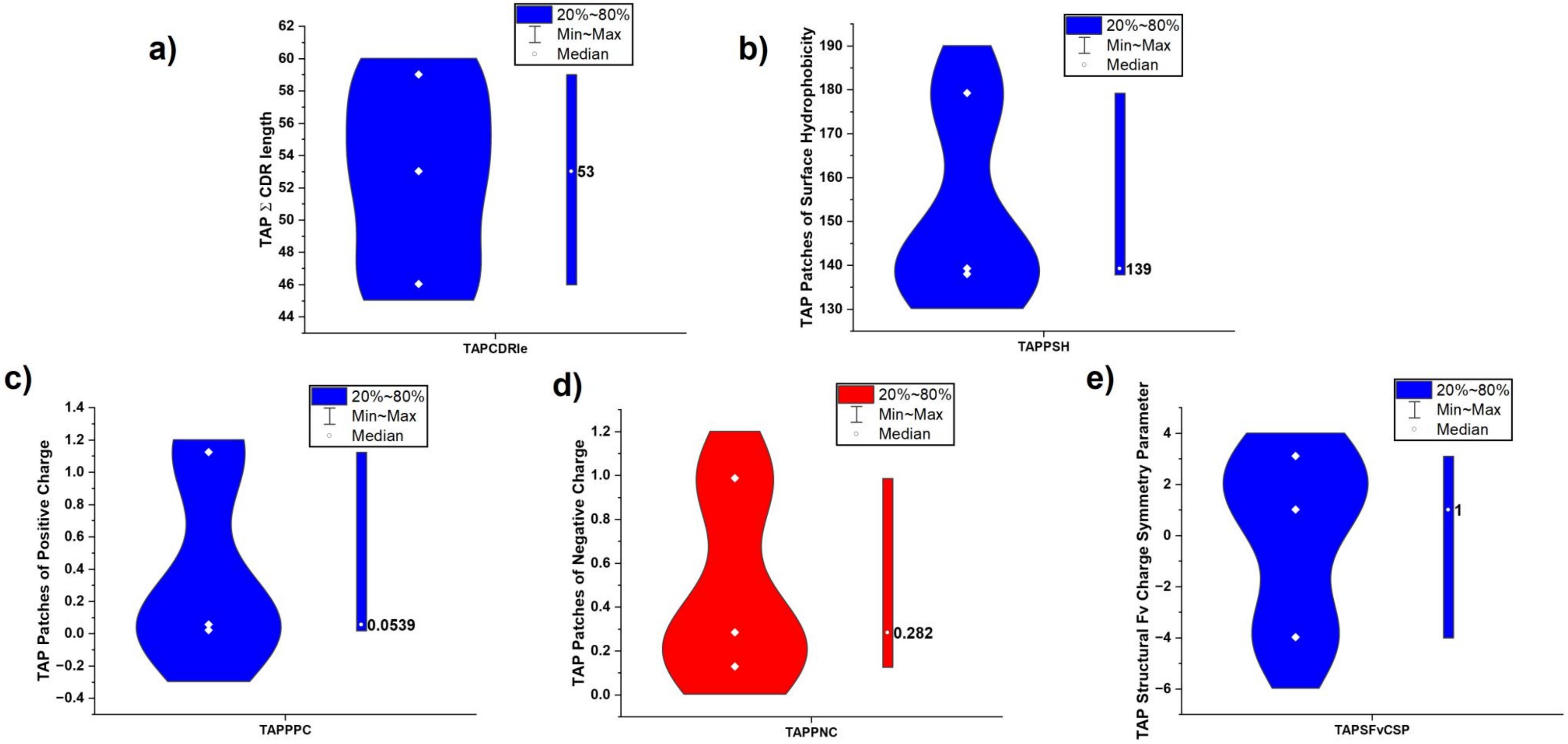

Figure S7. Characterisation of the antibody sequences at t=0 with the Therapeutic Antibody Profiler (TAP) webserver. Violin plots (left) and box and whisker diagrams (right) for: a) Total CDR length (TAPCDRle), b) Patches of Surface Hydrophobicity (TAPPSH), c) Patches of Positive Charge (TAPPPC), d) Patches of Negative Charge (TAPPNC) and e) Structural Fv Charge Symmetry Parameter (TAPSFvCSP). The box boundaries are the 20% and 80% percentiles. The median values are shown in bold. The bars span the range of the data. The diagrams are coloured in accord with the variable grouping in the Hierarchical Clustering dendrogram (Figure 4b). For reference, mAb2's respective TAP values are a) 53, b)139, c) 0.054, d) 0.987 and e) 1.

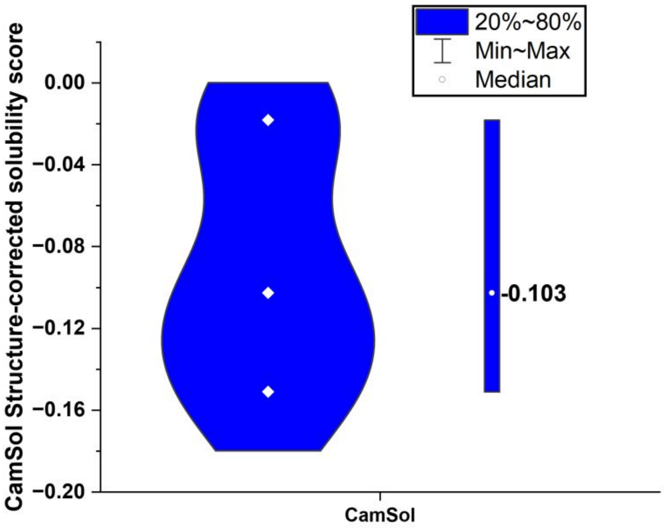

Figure S8. CamSol scores for mAbs 1-3 (structure-corrected using homology model structures) at t=0. The box boundaries = 20% and 80% percentiles. The median value is shown in bold. The bar spans the range of the data. The diagram is coloured in accord with the variable grouping in the Hierarchical Clustering dendrogram (Figure 4b). For reference, mAb2's structure-corrected CamSol score = -0.103.

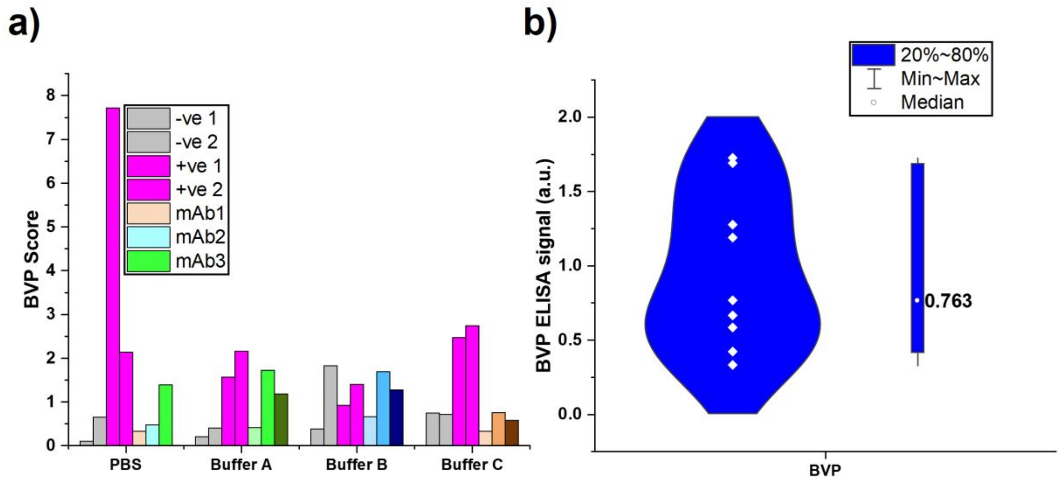

Figure S9. Characterisation of the formulation:mAb panel at t=0 by Baculovirus Particle ELISA. a) BVP score ascertained from the ELISA analysis of the different antibody formulations (Supplementary Methods). -ve and +ve correspond to negative (low binding) and positive (high binding) control molecules, respectively. b) Violin plot (left) and box and whisker diagram (right) showing the distribution of the retention time data. The box boundaries = 20% and 80% percentiles. The median value is shown in bold. The bar spans the range of the data and the data coloured in accord with the variable grouping in the Hierarchical Clustering dendrogram (Figure 4b).

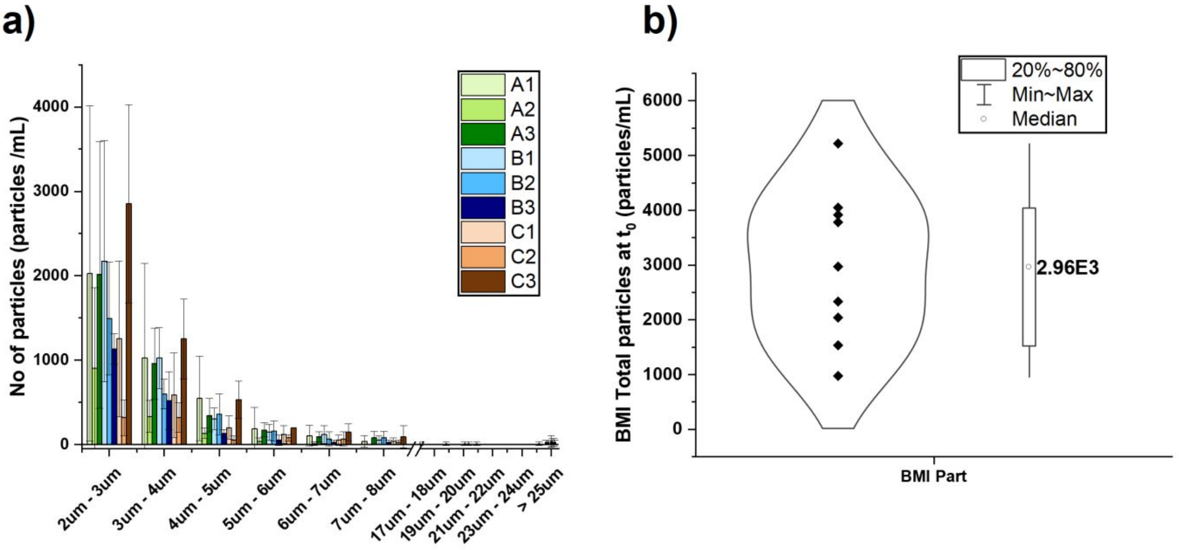

Figure S10. Characterisation of the number of particles in each formulation:mAb at t=0 by Background Membrane Imaging (BMI). a) Histogram of the number of particles in each formulation greater than 2 µm in diameter. b) Violin plot (left) and box and whisker diagram (right) showing the distribution of the total number of particles with a diameter greater than 2 µm within each formulation (obtained by summing the bars shown in a, for each respective formulation). The box boundaries are the 20% and 80% percentiles. The median value is shown in bold. The bar spans the range of the data. The diagram is colourless, due to its subsequent exclusion from the final suite of assay variables.

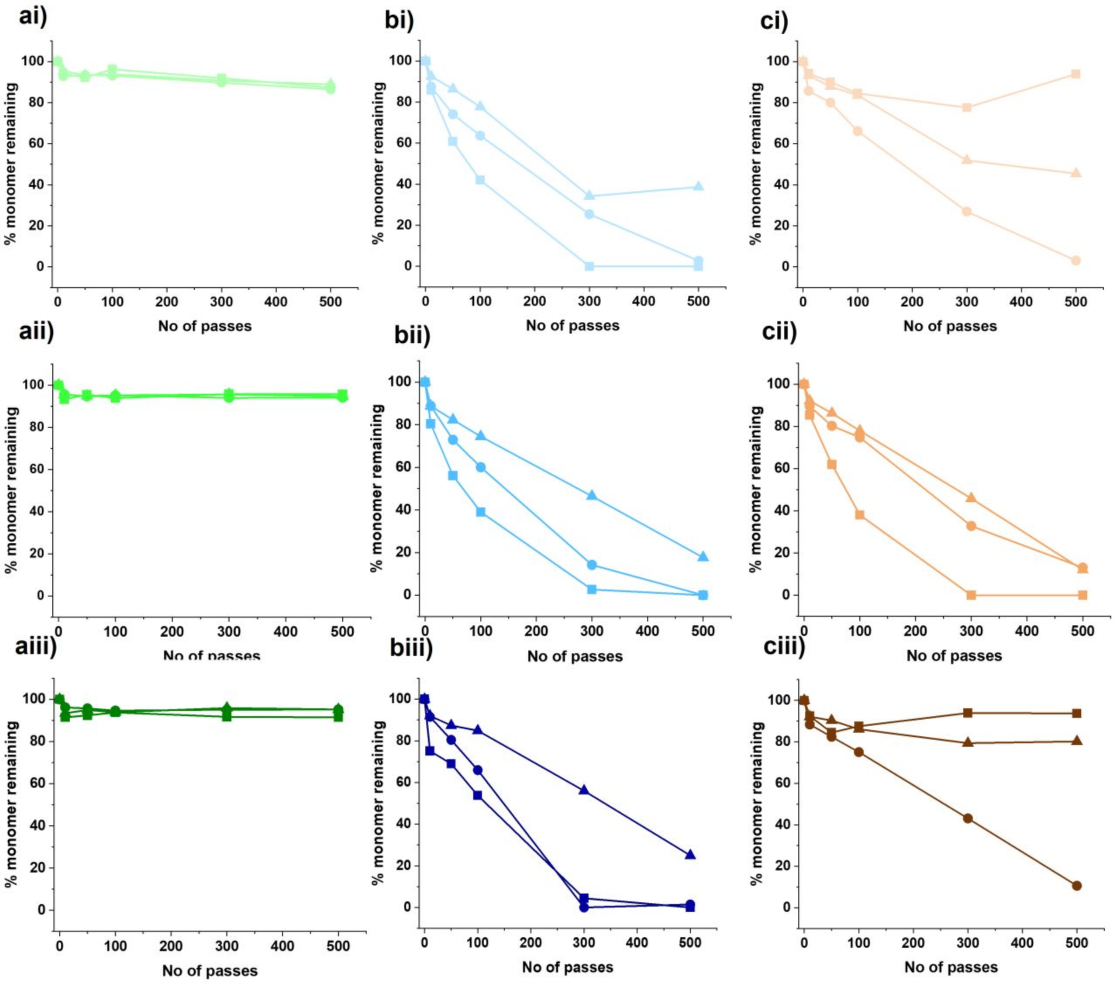

Figure S11. Characterising % monomer remaining in the formulation:mAb panel by HP-SEC (Methods) following Extensional Flow Device-induced aggregation. The mAbs: i) mAb1, ii) mAb2 and iii) mAb3, were stressed at a concentration of 0.25 (squares), 0.5 (circles) and 1 mg/mL (triangles) for 0–500 passes in Buffers A (histidine-arginine, a), B (histidine-sucrose, b) and C (sodium citrate, c). The lines connecting the points are guides for the eye, not fits to the data. n = 1 biological repeat.

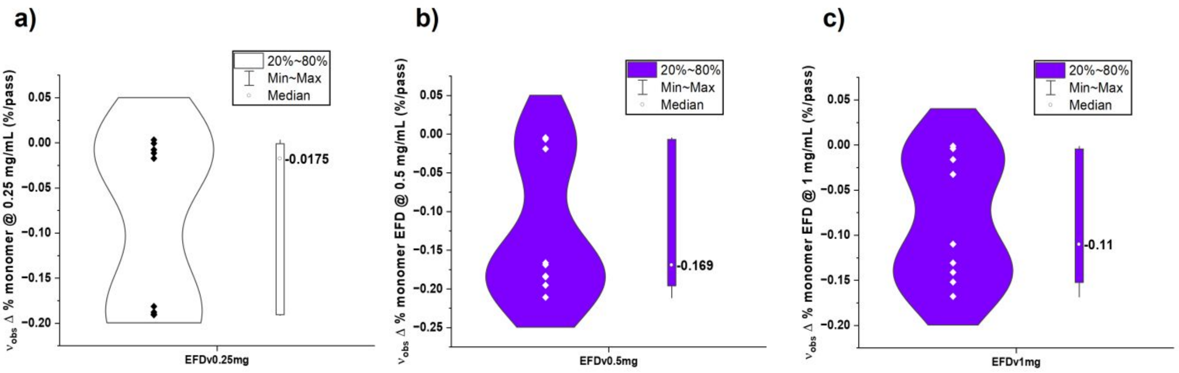

Figure S12. Violin and box and whisker plots of the observed rates of change in % monomer following stress in the EFD (EFDv) at a) 0.25 mg/mL, b) 0.5 mg/mL and c) 1 mg/mL. Negative values indicate the loss of monomer from solution. The data are obtained from the SLOPE function being applied to the data in Figure S11, using Microsoft Excel. The box boundaries are 20% and 80% percentiles. The median values are shown in bold. The diagrams are coloured in accord with the variable grouping in the Hierarchical Clustering dendrogram (Figure 4b), except for Figure S12a, due to its subsequent exclusion from the final suite of assay variables.

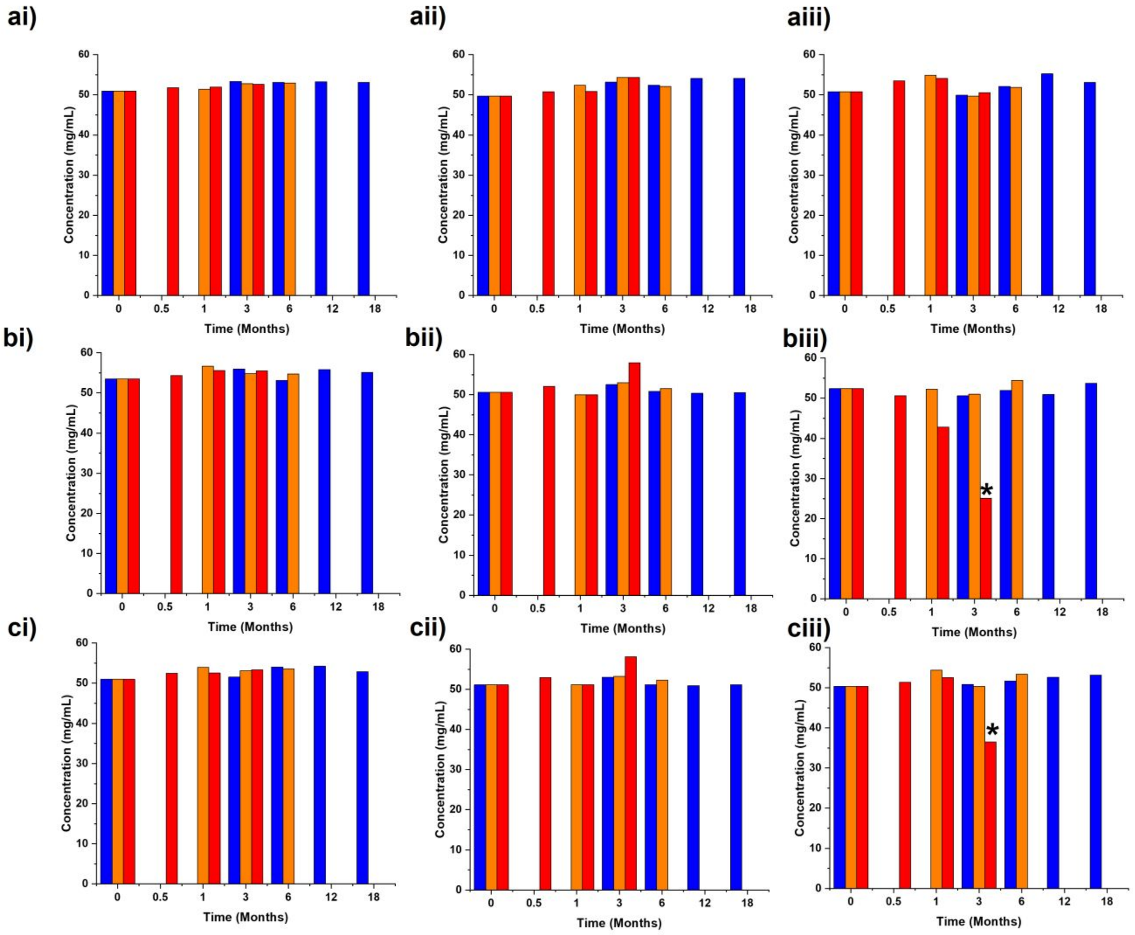

Figure S13. Monitoring the concentration of soluble protein over time. a–c) Measurement of protein concentration using the Trinean DropSense instrument across the accelerated and storage stability study (Methods) at 5 °C (blue), 25 °C (orange) and 40 °C (red) for a) mAb1, b) mAb2 and c) mAb3 in Buffer A (i), Buffer B (ii) and Buffer C (iii), respectively. NB precipitation was observed for the C2 and C3 samples at 3 months, 40 °C timepoints (biii and ciii, respectively), as denoted by the *. The average and s.d. across the measurements shown above, are in Table S2.

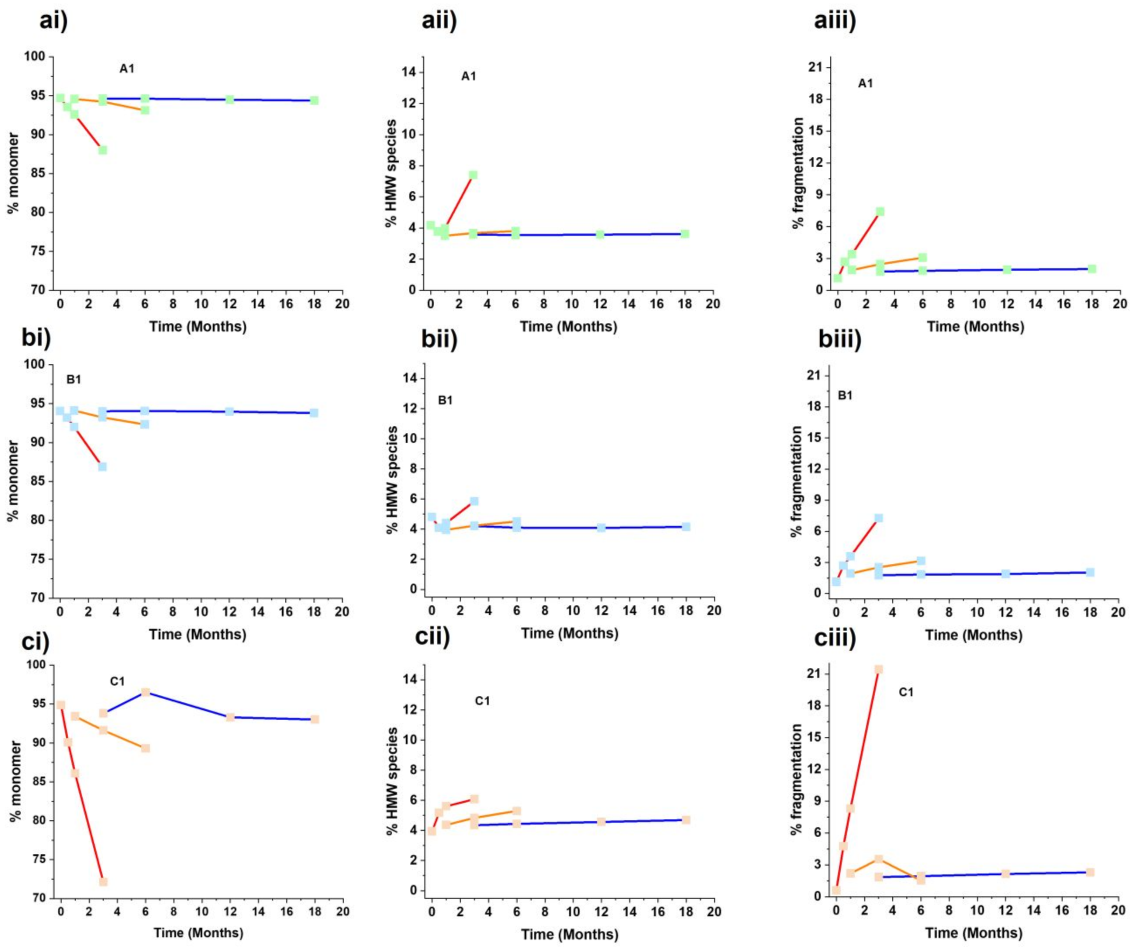

Figure S14. Accelerated and storage stability data for mAb1 in Buffers A (histidine-arginine, a) B (histidine-sucrose, b) and C (sodium citrate, c), characterised by HP-SEC. i) % monomer versus time after storage at 5 °C, 25 °C and 40 °C. ii) % Higher molecular weight (HMW) species versus time after storage at 5 °C, 25 °C and 40 °C. iii) % fragmentation (frag) versus time after storage at 5 °C, 25 °C and 40 °C. The lines through the points at 5 °C (blue), 25 °C (orange) and 40°C (red) are to guide the eye, not fits to the data.

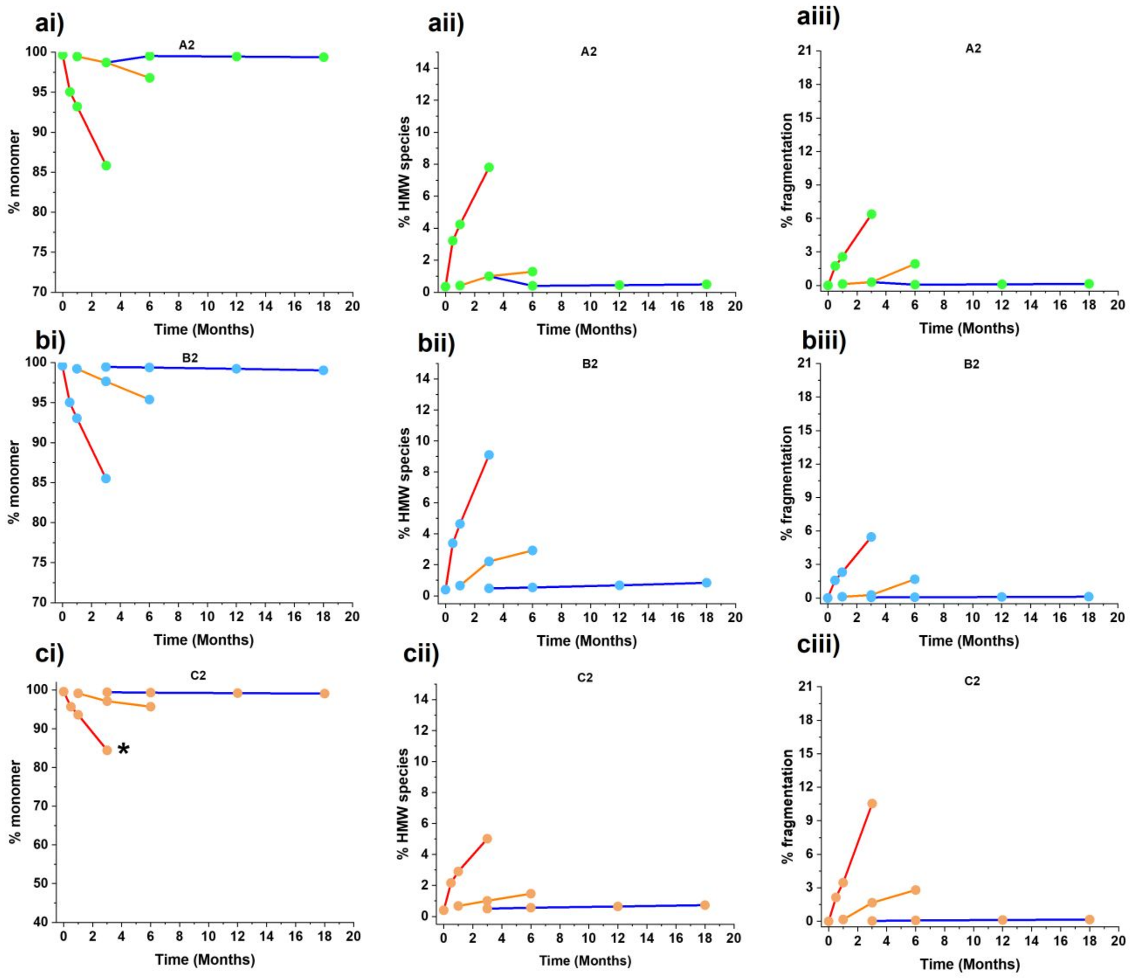

Figure S15. Accelerated and storage stability data for mAb2 in Buffers A (histidine-arginine, a) B (histidine-sucrose, b) and C (sodium citrate, c), characterised by HP-SEC. i) % monomer versus time after storage at 5 °C, 25 °C and 40 °C. ii) % Higher molecular weight (HMW) species versus time after storage at 5 °C, 25 °C and 40 °C. iii) % fragmentation (frag) versus time after storage at 5 °C, 25 °C and 40 °C. The lines through the points at 5 °C (blue), 25 °C (orange) and 40°C (red) are to guide the eye, not fits to the data. The * denotes precipitation in the initial sample, corroborated by measurement of the soluble protein concentration (Figure S13), thus the point was removed from the SLOPE analysis.

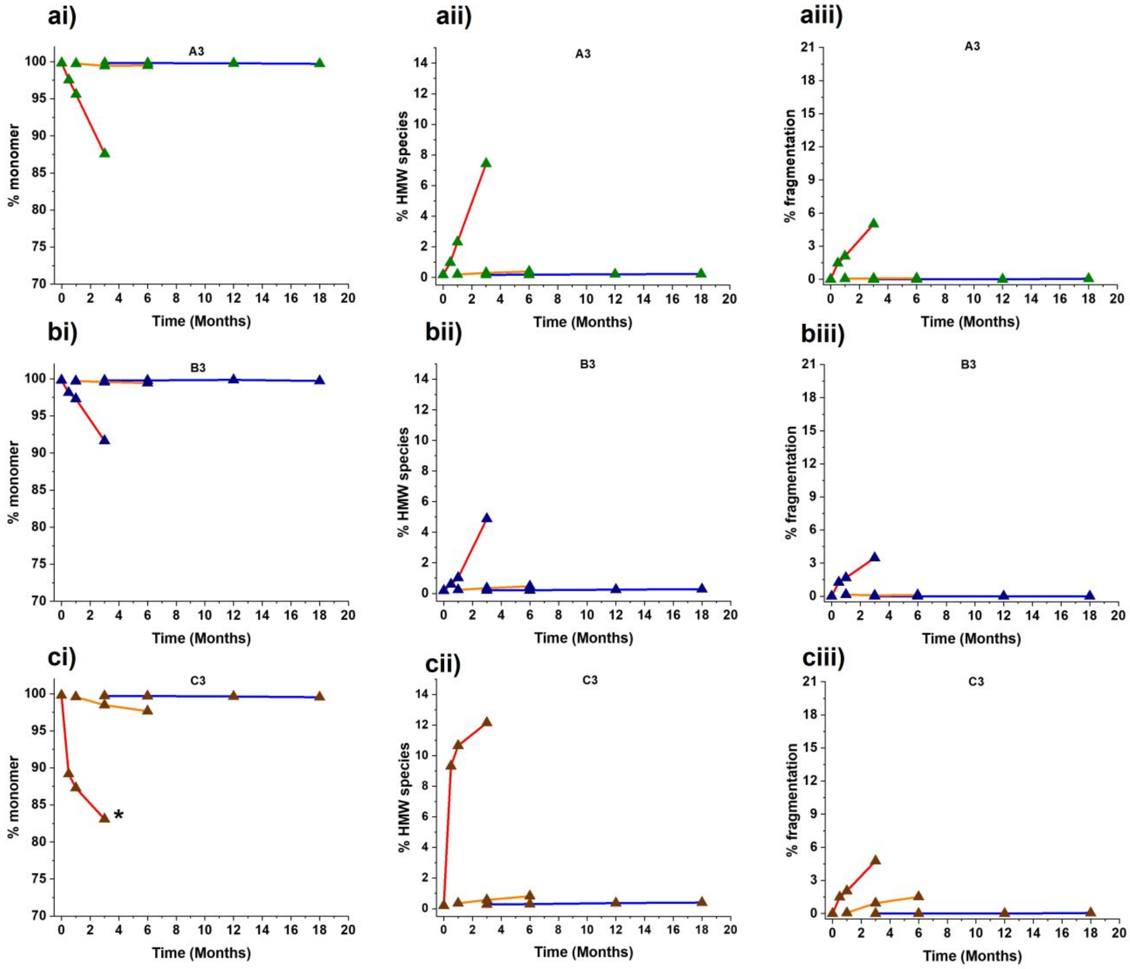

Figure S16. Accelerated and storage stability data for mAb3 in Buffers A (histidine-arginine, a) B (histidine-sucrose, b) and C (sodium citrate, c), characterised by HP-SEC. % monomer versus time after storage at 5 °C, 25 °C and 40 °C. ii) % Higher molecular weight (HMW) species versus time after storage at 5 °C, 25 °C and 40 °C. iii) % fragmentation (frag) versus time after storage at 5 °C, 25 °C and 40 °C. The lines through the points at 5 °C (blue), 25 °C (orange) and 40°C (red) are to guide the eye, not fits to the data. The * denotes precipitation in the initial sample, corroborated by measurement of the soluble protein concentration (Figure S13), thus the point was removed from the SLOPE analysis.

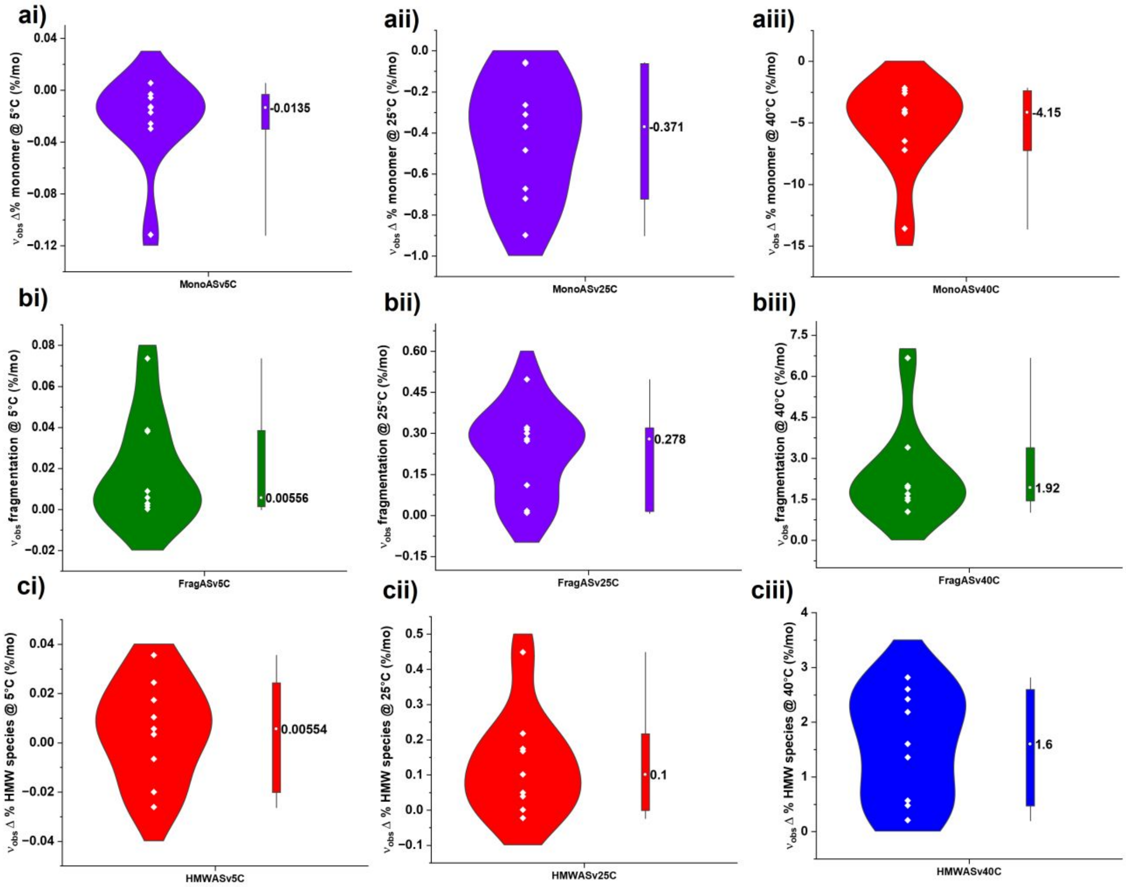

Figure S17. Violin and box and whisker plots of the observed rates of change in a) % monomer (MonoASv), b) fragmentation (FragASv) and c) % higher molecular weight species (HMWASv) at i) 5 °C, ii) 25 °C and iii) 40 °C. Negative values (for monomer) indicate loss of material and positive values for higher molecular weight and fragmented species indicate their accumulation. The data were obtained from the SLOPE function being applied to the data in Figures S14-S16 in Microsoft Excel. Box boundaries show 20% and 80% percentiles, median values are shown in bold and the bars span the range of the data. The diagrams are coloured in accord with the variable grouping in the Hierarchical Clustering dendrogram (Figure 4b).

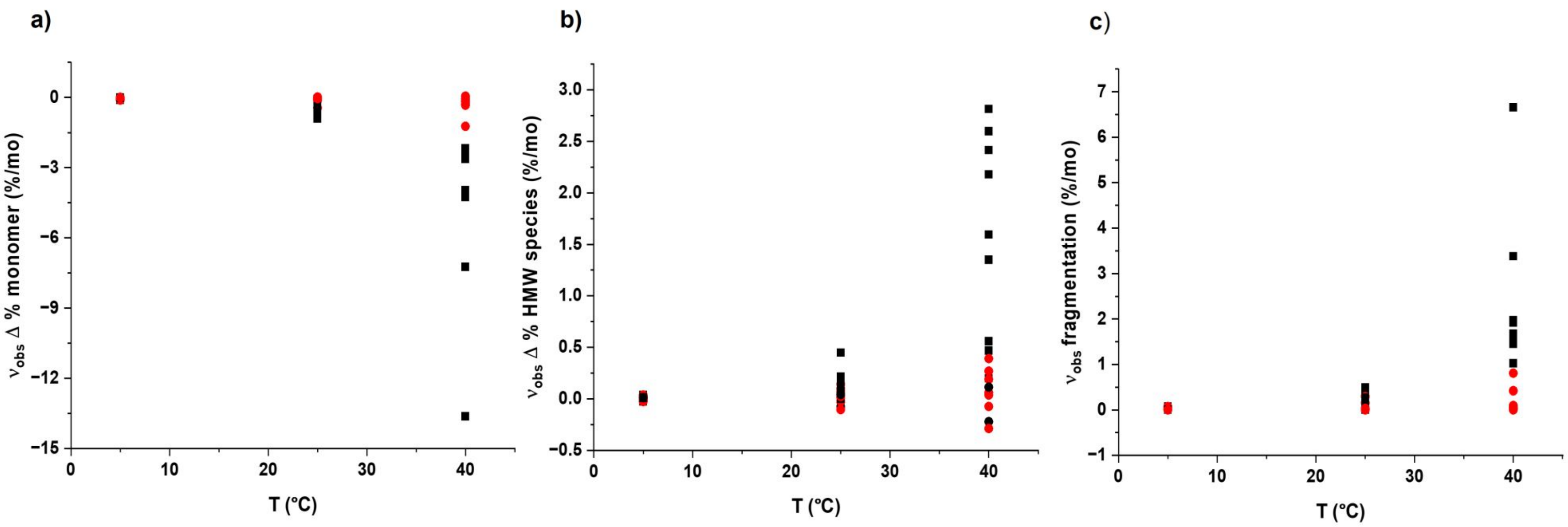

Figure S18. Dependence of the observed rate of change in a) % monomer, b) % HMW species and c) fragmentation as a function of temperature. Negative values (for monomer) indicate loss of material and positive values for higher molecular weight and fragmented species indicating their accumulation. The predicted data (red points) were generated assuming a 10 °C increase in temperature leads to a doubling of the observed rate of reaction, based on experiment-derived data across the stability study at 5 °C, 25 °C and 40 °C . The experimental rates obtained in the storage stability study, from Figure S17, are shown in black.

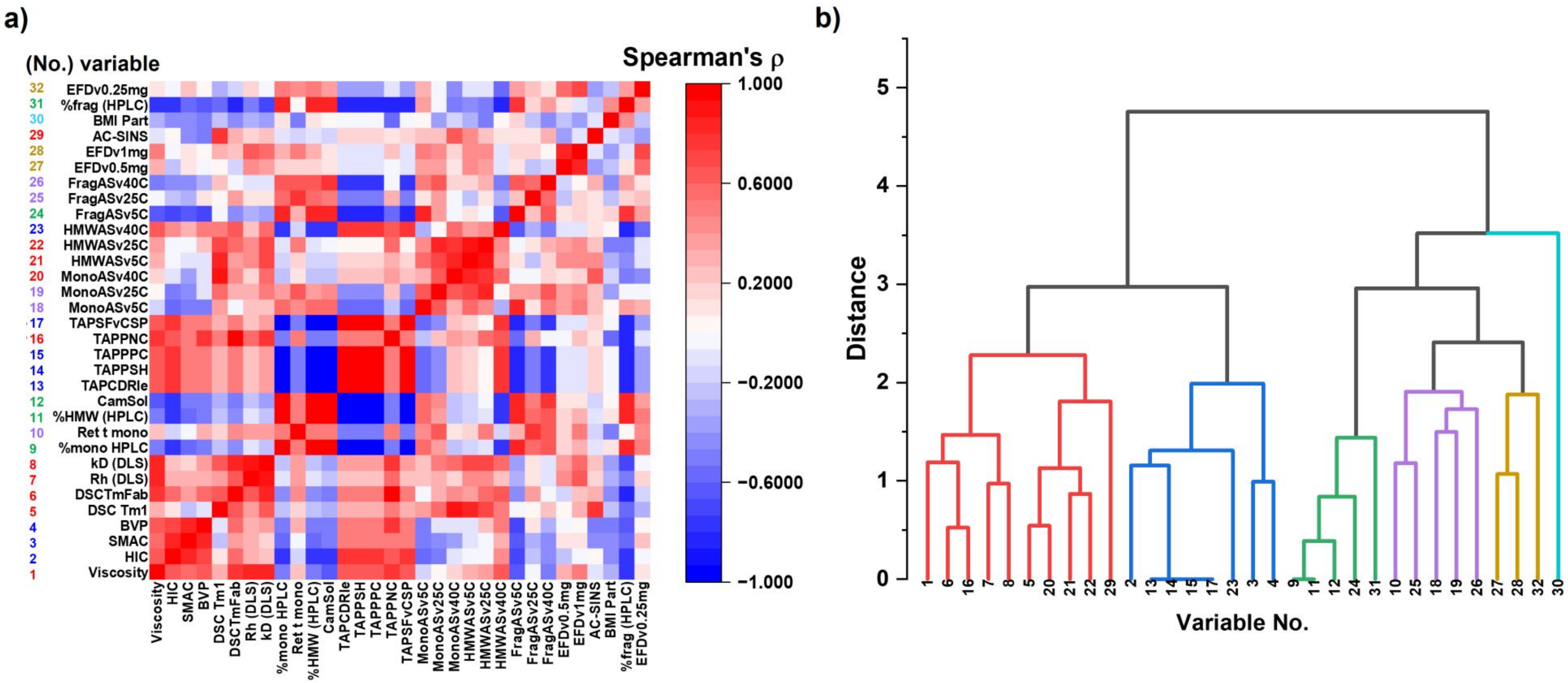

Figure S19. a) Heatmap of Spearman's rank correlation coefficients (ρ) and b) Dendrogram of assay variables derived from Hierarchical Clustering analysis. The initial set of 32 assay variables were input into the Spearman's rank correlation analysis (a), forming six distinct assay clusters (b). Clusters are coloured and labelled in both diagrams. To guide the reader, of the five variables in the purple branch, the observed rates of change in % monomer following long-term storage at 5 °C and 25 °C are variables 18 and 19, respectively. EFD assay metrics are shown in gold (variables 27, 28 and 32). Numerical identification numbers are defined in Tables 3–5.

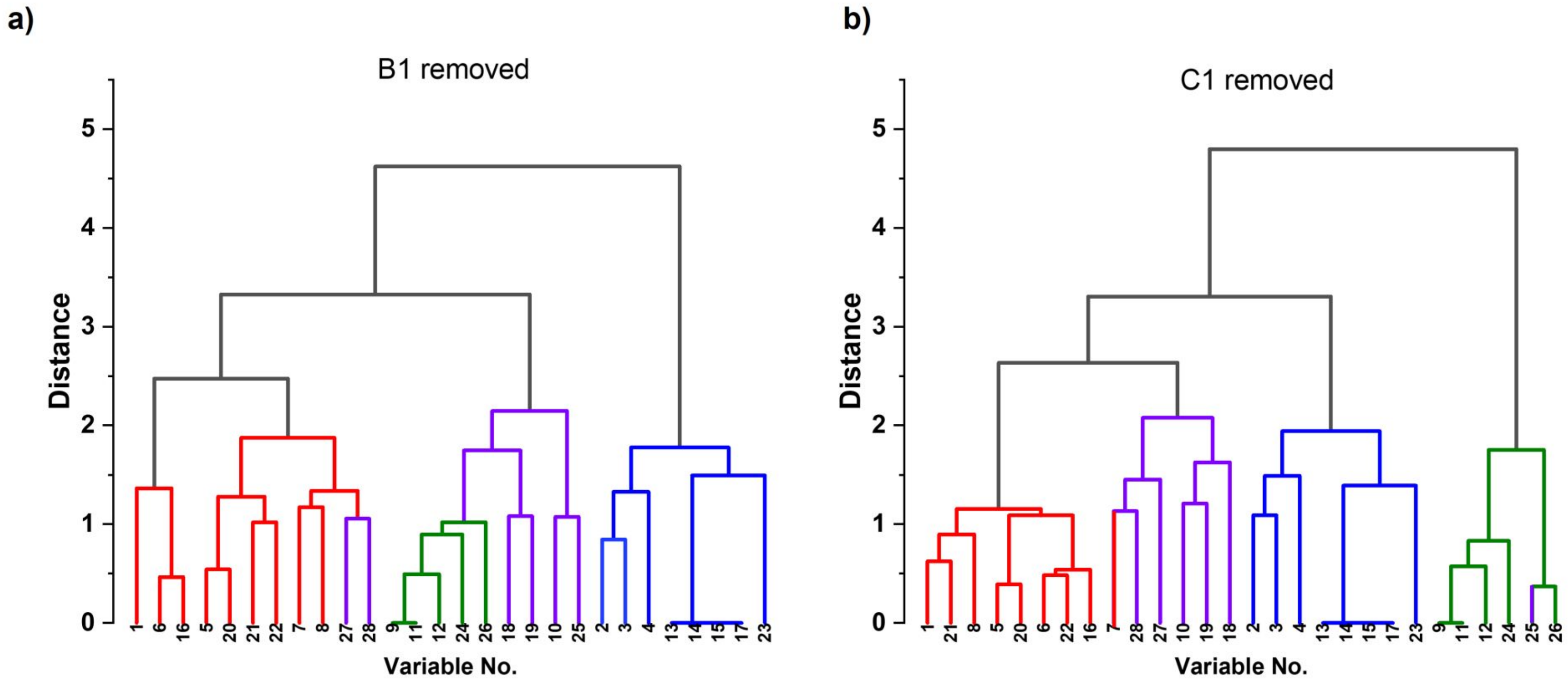

Figure S20. Testing the limit of assay grouping correlations by removing formulations from the dataset. The final set of 28 assay variables from Figure 4 were used as the input for the sensitivity analysis. Each formulation was removed in turn from the dataset, which was then re-ranked, with Spearman's rank correlation and Hierarchical Clustering analysis then performed. Dendrograms shown are the assay variables derived from Hierarchical Clustering analysis when formulations B1 a) or C1 b) are removed from the panel. Clusters are coloured and labelled in both diagrams in the same scheme as Figure 4b. Numerical identifiers for each variable can be found in Tables 1–3.

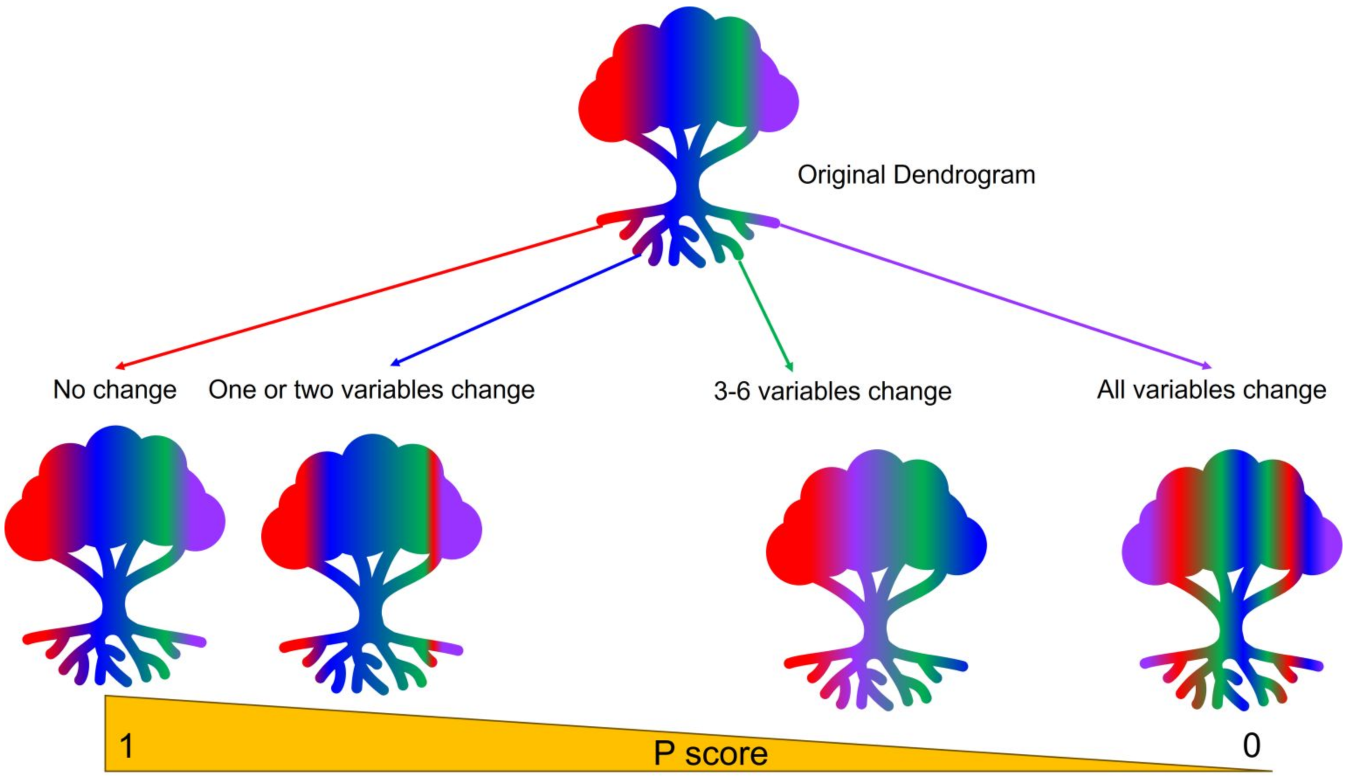

Figure S21. Schematic of hierarchical clustering robustness analysis using the method of Lu et al.[9] The total number of times a variable paired with its immediate neighbour was counted in each iteration of the analysis, then divided by the total number of iterations (10). Values (termed P) close to 1 reflected the most robustly clustered variables. When performed across the original set of 32 variables, a median P value of 0.9 was obtained. P =1 for 12/32 variables (i.e., no change in pairing), P= 0.9 for 6/32 variables (one change), P = 0.8 for 10/32 variables (two changes), with just 4/32 changing three (P = 0.7,1 var.), four (P= 0.6, 2 var.) or six (P = 0.4,1 var.) times, respectively. When re-performed on the best-clustered 28 variables, the median P value from the analysis = 0.9. P = 1 for 10/28 variables, P= 0.9 for 5/28 variables, P = 0.8 for 6/28 variables, with the remaining seven changing three (P = 0.7, 2 var.), four (P = 0.6, 4 var.) or six (P = 0.4, 1 var.) times, respectively.

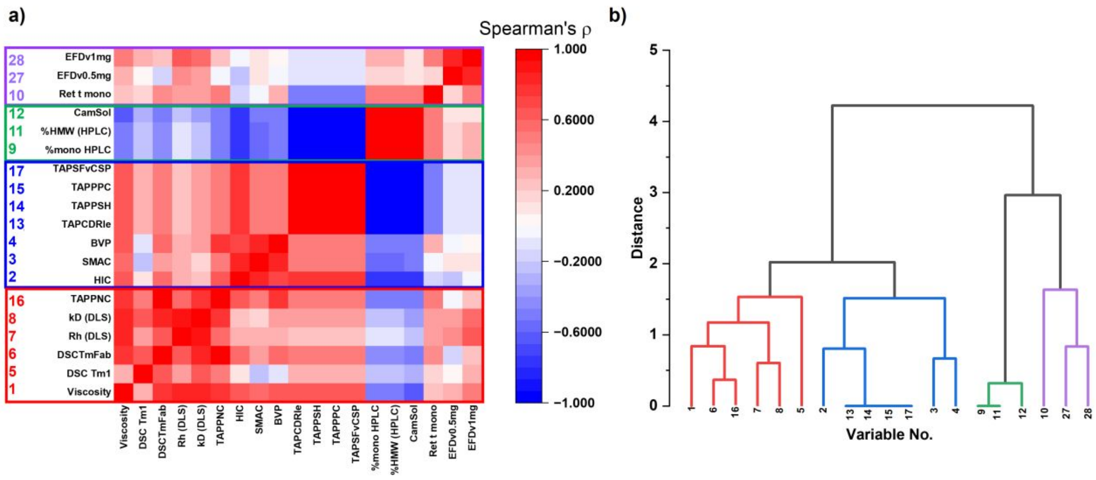

Figure S22. Hierarchical clustering in the absence of group V (AS and LTS data). (a) Heatmap of Spearman's rank correlation coefficients (ρ) from the 19 developability assay variables which do not derive from the 5 °C and 25 °C long-term storage stability datasets, nor the 40 °C accelerated stability dataset. (b) Dendrogram of the assay variables. Assay groupings are the same as those in Figure 4, with numerical identification numbers are defined in Tables 3-5.

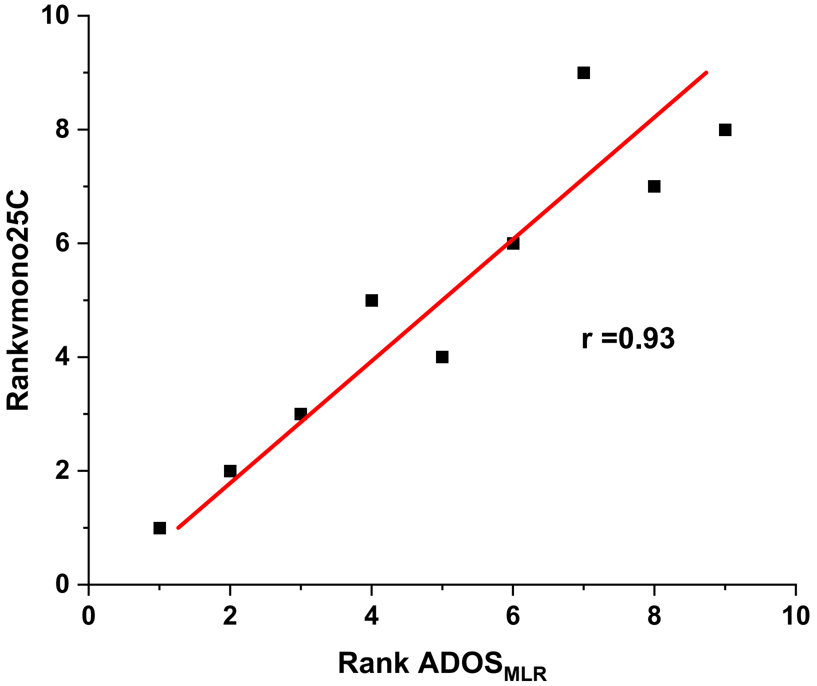

Figure S23. Multiple Linear Regression (MLR) helps optimise the ADOS to predict 25 °C **storage stability.** Correlation of the ranked ADOS output, with branches weighted using Multiple Linear Regression, shows that this method can optimise the correlation against storage stability.

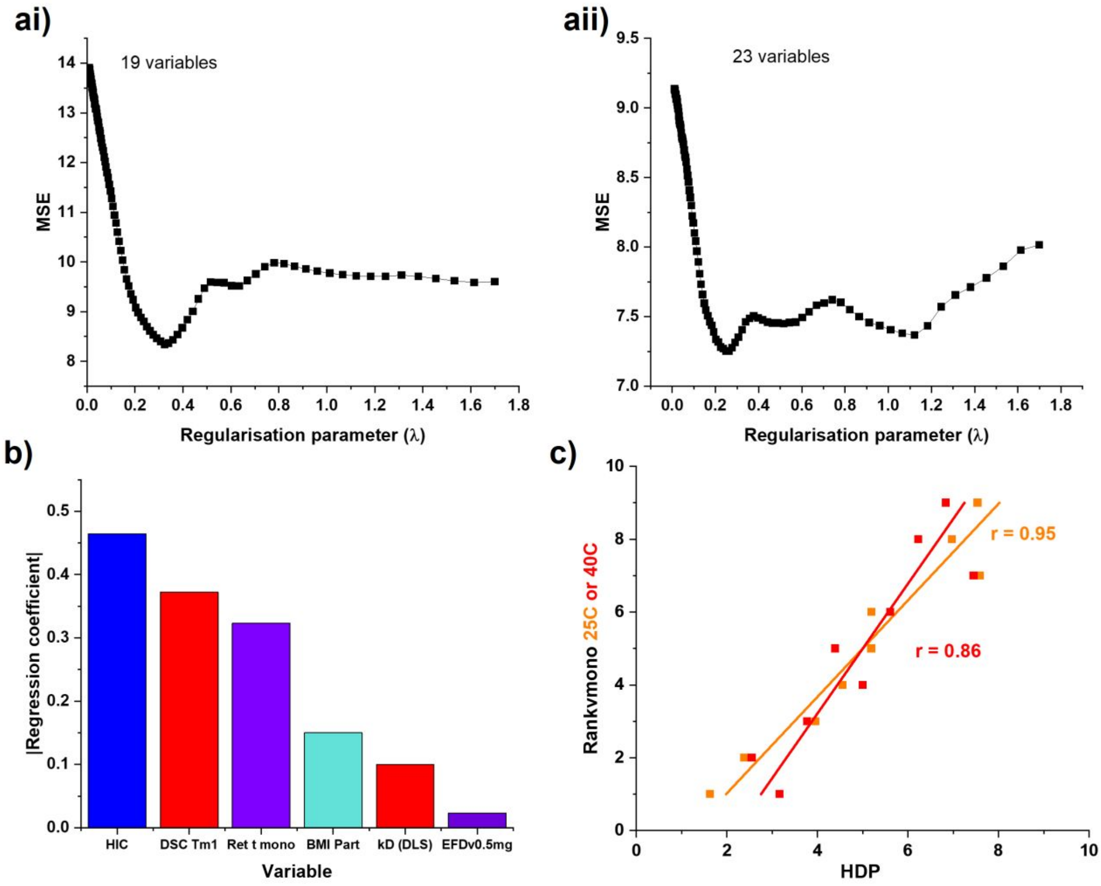

Figure S24. LASSO regression effectively constrains the final number of assay variables used to predict storage stability. a) Evolution of the mean square error (MSE) as a function of the regularisation parameter (λ), calculated by cross-validation, in the absence of the variables derived from the long-term or accelerated stability study and in the absence (i) and presence (ii) of difficult to cluster variables. b) For the latter dataset, the six variables selected by LASSO include all of those from Figure 6bi, with total particle count from BMI (BMI Part, variable 30, cyan) joining this set. c) Plot of Holistic Developability Parameter (HDP) scores (derived using a subset of 6 variables identified by LASSO analysis of the data) obtained for the nine formulation:mAbs versus observed rate of change in % monomer at either 25 °C (orange) or 40 °C (red). For the 25 °C example, the addition of the number of particles measured using BMI (variable 30) marginally improves the fit, compared to the five selected in Figure 6bi) (Pearson's r = 0.95 vs 0.92). Conversely, the only variable selected by LASSO to predict ranked stability at 40 °C (Pearson's r = 0.86) is the 1st apparent Tm from DSC (variable 5). Despite LASSO having no knowledge of the Hierarchical Clustering analysis (where this variable clusters with Rankvmono40C (variable 20)), its selection both validates the clustering data and the observation that the behaviour of antibodies at elevated temperatures may not correlate with room temperature assays.[10]

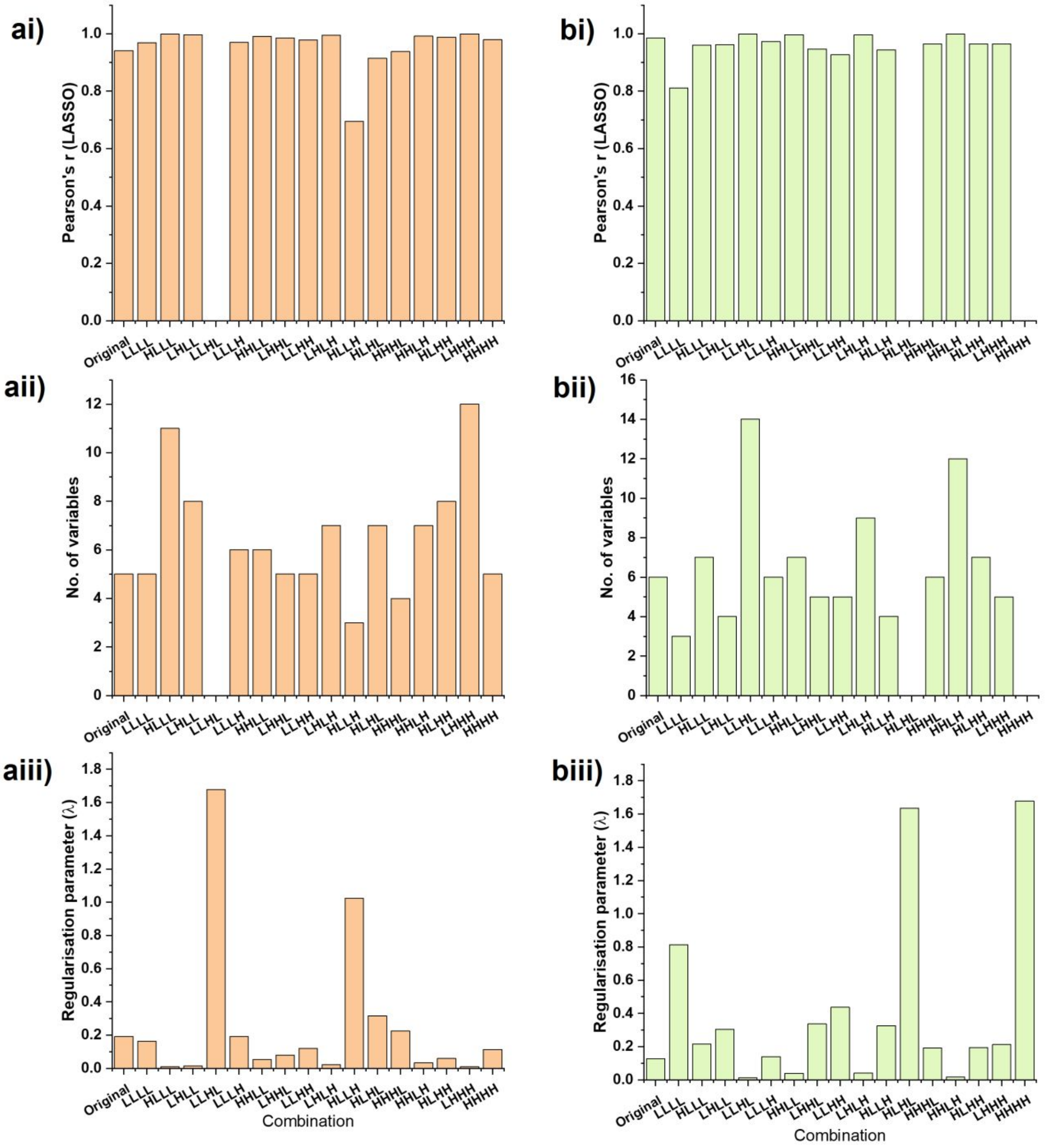

Figure S25. Robustness analysis of LASSO regression based on various combinations of ranked % change in monomer at 25 °C data. For each combination (where H and L signify the highest and lowest degradation rates based on the calculated error, the variable datasets were the 19 best-clustered variables (a) or the 23 variables (b) neither of which inclide Group V (kinetic stability data). Robustness was assessed by i) Pearson's r, ii) the number of variables selected by the model and iii) the magnitude of the regularisation parameter selected from the cross-validation analysis in XLSTAT. The median values from these analyses are: ai) 0.98, bi) 0.96, aii and bii) 6 aiii) 0.114 and biii) 0.212.

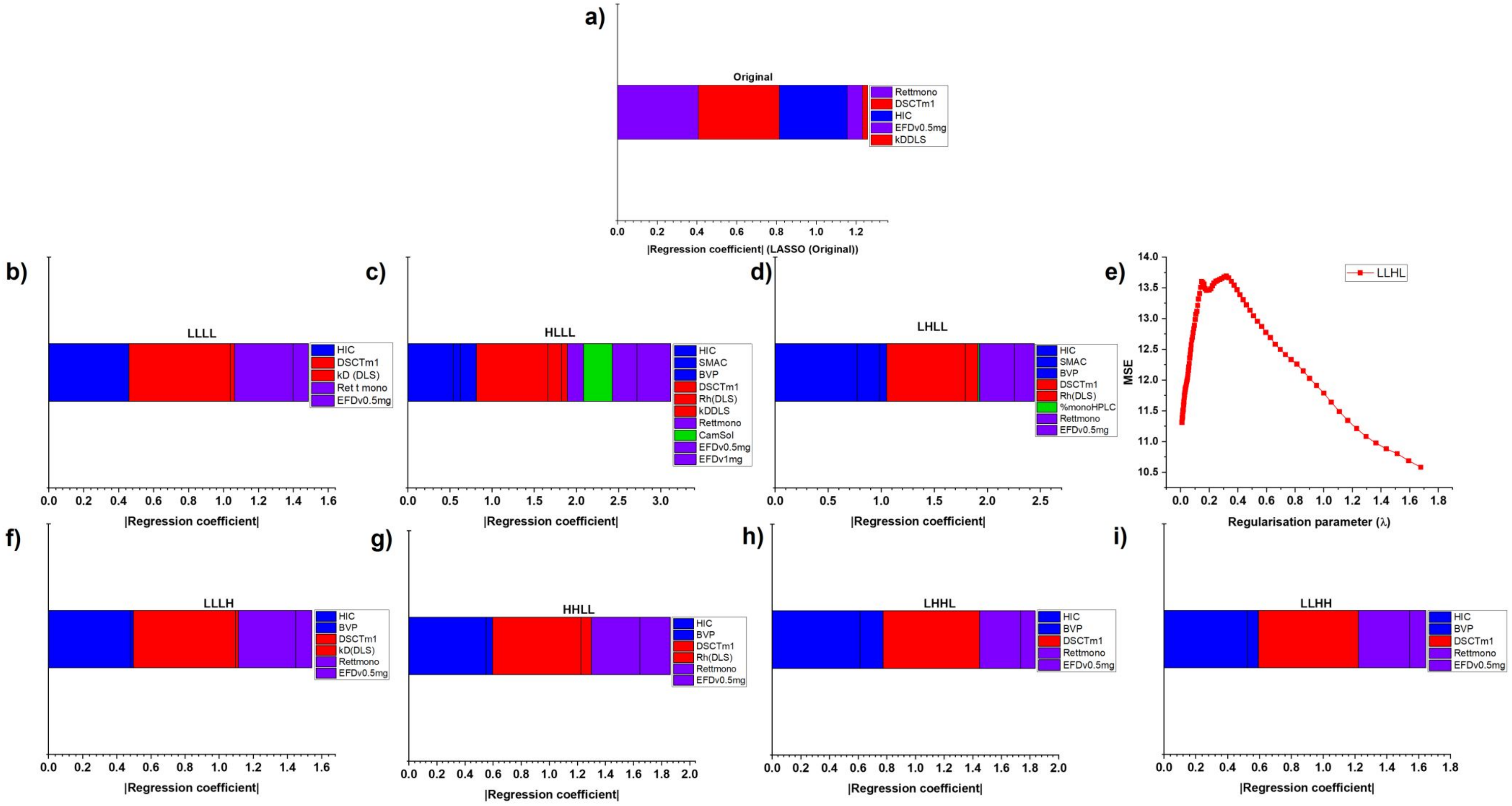
a)
Original
Rettmono
DSCTm1
HIC
EFDv0.5mg
kDDLS
|Regression coefficient| (LASSO (Original))
b)
LLLL
HIC
DSCTm1
kD (DLS)
Ret t mono
EFDv0.5mg
|Regression coefficient|
c)
HLLL
HIC
SMAC
BVP
DSCTm1
Rh(DLS)
kDDLS
Rettmono
CamSol
EFDv0.5mg
EFDv1mg
|Regression coefficient|
d)
LHLL
HIC
SMAC
BVP
DSCTm1
Rh(DLS)
%monoHPLC
Rettmono
EFDv0.5mg
|Regression coefficient|
e)
LLHL
MSE
Regularisation parameter (λ)
f)
LLLH
HIC
BVP
DSCTm1
kD(DLS)
Rettmono
EFDv0.5mg
|Regression coefficient|
g)
HHLL
HIC
BVP
DSCTm1
Rh(DLS)
Rettmono
EFDv0.5mg
|Regression coefficient|
h)
LHHL
HIC
BVP
DSCTm1
Rettmono
EFDv0.5mg
|Regression coefficient|
i)
LLHH
HIC
BVP
DSCTm1
Rettmono
EFDv0.5mg
|Regression coefficient|

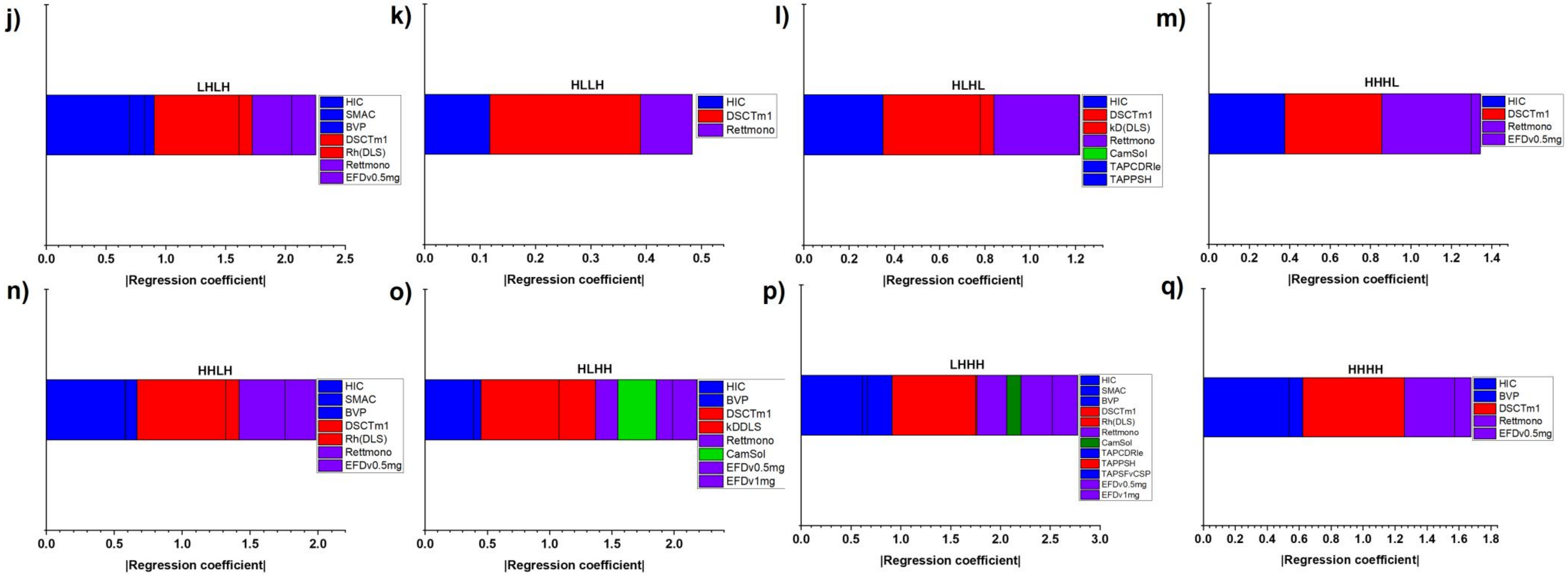

Figure S26. LASSO regression coefficients for the best clustered variables, starting with the original ranking (a) and then using the re-ranked observed rate of change in % monomer at 25 °C (b–q). Variables are coloured according to assay cluster from Figure 4. The four letters represent the lower (L) or upper (H) limits of these rates for formulations A1, B1, A2 and C3 in each respective position. In e, the regularisation parameters sampled during the LASSO regression did not find an energetic minimum during the cross-validation, resulting in no parameters selected and thus a nul fit was obtained for this combination.

Table S1. pH and osmolality values of the final formulation:mAb samples. Osmolality = N = 3 technical replicates, error bar = s.d.

Formulation:mAb	A1	A2	A3	B1	B2	B3	C1	C2	C3
pH	6.16	6.07	6.03	6.11	5.99	6.00	4.93	5.05	4.96
Osmolality (mOs/kg)	369±5	369±2.3	369±1	298±3	319±6.7	318±11	64±0.6	96±1.5	71±2

Table S2. Average soluble protein concentration of the nine formulation:mAb samples, measured over the length of the AS and LTS studies (N =11, per formulation). The only samples which showed a significant decrease in soluble protein were C2 and C3, after incubation at 40°C for 3 months (see Figure S13), accounting for the larger standard deviation observed for these formulations (highlighted in red).

Sample	[Protein] t=0 (mg/mL)	Average [Protein] (mg/mL)	s.d.	2 s.d.
All formulations (A1–C3)	51.2	52.1	1.7	3.3
A1	51.0	52.5	0.8	1.6
A2	53.5	55.1	0.9	1.8
A3	51.0	53.0	1.0	2.1
B1	49.7	52.6	1.6	3.2
B2	50.6	51.8	2.3	4.6
B3	51.1	52.4	2.1	4.2
C1	50.8	52.3	2.0	3.9
C2	52.4	48.7	8.4	16.8
C3	50.3	50.7	4.9	9.75

Supplementary References

(1) Jain, T.; Sun, T.; Durand, S.; Hall, A.; Houston, N. R.; Nett, J. H.; Sharkey, B.; Bobrowicz, B.; Caffry, I.; Yu, Y.; Cao, Y.; Lynaugh, H.; Brown, M.; Baruah, H.; Gray, L. T.; Krauland, E. M.; Xu, Y.; Vásquez, M.; Wittrup, K. D. Biophysical Properties of the Clinical-Stage Antibody Landscape. *Proceedings of the National Academy of Sciences* **2017**, *114* (5), 944–949. https://doi.org/10.1073/pnas.1616408114.

(2) Sule, S. V; Dickinson, C. D.; Lu, J.; Chow, C.-K.; Tessier, P. M. Rapid Analysis of Antibody Self-Association in Complex Mixtures Using Immunogold Conjugates. *Mol Pharm* **2013**, *10* (4), 1322–1331. https://doi.org/10.1021/mp300524x.

(3) Shih, P.; Kirsch, J. F.; Holland, D. R. Thermal Stability Determinants of Chicken Egg-white Lysozyme Core Mutants: Hydrophobicity, Packing Volume, and Conserved Buried Water Molecules. *Protein Science* **1995**, *4* (10), 2050–2062. https://doi.org/10.1002/pro.5560041010.

(4) Raybould, M. I. J.; Marks, C.; Krawczyk, K.; Taddese, B.; Nowak, J.; Lewis, A. P.; Bujotzek, A.; Shi, J.; Deane, C. M. Five Computational Developability Guidelines for Therapeutic Antibody Profiling. *Proceedings of the National Academy of Sciences* **2019**, *116* (10), 4025–4030. https://doi.org/10.1073/pnas.1810576116.

(5) Willis, L. F.; Kumar, A.; Jain, T.; Caffry, I.; Xu, Y.; Radford, S. E.; Kapur, N.; Vásquez, M.; Brockwell, D. J. The Uniqueness of Flow in Probing the Aggregation Behavior of Clinically Relevant Antibodies. *Engineering Reports* **2020**, *2* (5), 1–13. https://doi.org/10.1002/eng2.12147.

(6) Sormanni, P.; Aprile, F. A.; Vendruscolo, M. The CamSol Method of Rational Design of Protein Mutants with Enhanced Solubility. *J Mol Biol* **2015**, *427* (2), 478–490. https://doi.org/10.1016/j.jmb.2014.09.026.

(7) Pettersen, E. F.; Goddard, T. D.; Huang, C. C.; Couch, G. S.; Greenblatt, D. M.; Meng, E. C.; Ferrin, T. E. UCSF Chimera--a Visualization System for Exploratory Research and Analysis. *J Comput Chem* **2004**, *25* (13), 1605–1612. https://doi.org/10.1002/jcc.20084.

(8) Dobson, C. L.; Devine, P. W. A.; Phillips, J. J.; Higazi, D. R.; Lloyd, C.; Popovic, B.; Arnold, J.; Buchanan, A.; Lewis, A.; Goodman, J.; van der Walle, C. F.; Thornton, P.; Vinall, L.; Lowne, D.; Aagaard, A.; Olsson, L.-L.; Ridderstad Wollberg, A.; Welsh, F.; Karamanos, T. K.; Pashley, C. L.; Iadanza, M. G.; Ranson, N. A.; Ashcroft, A. E.; Kippen, A. D.; Vaughan, T. J.; Radford, S. E.; Lowe, D. C. Engineering the Surface Properties of a Human Monoclonal Antibody Prevents Self-Association and Rapid Clearance in Vivo. *Sci Rep* **2016**, *6* (November), 38644. https://doi.org/10.1038/srep38644.

(9) Lu, Y.; Phillips, C. A.; Langston, M. A. A Robustness Metric for Biological Data Clustering Algorithms. *BMC Bioinformatics* **2019**, *20* (S15), 503. https://doi.org/10.1186/s12859-019-3089-6.

(10) Wälchli, R.; Vermeire, P.-J.; Massant, J.; Arosio, P. Accelerated Aggregation Studies of Monoclonal Antibodies: Considerations for Storage Stability. *J Pharm Sci* **2020**, *109* (1), 595–602. https://doi.org/10.1016/j.xphs.2019.10.048.
